# Surface characteristics and bacterial adhesion of endodontic cements

**DOI:** 10.1007/s00784-022-04655-y

**Published:** 2022-08-05

**Authors:** Andreas Koutroulis, Håkon Valen, Dag Ørstavik, Vasileios Kapralos, Josette Camilleri, Pia Titterud Sunde

**Affiliations:** 1 Section of Endodontics, Institute of Clinical Dentistry, Faculty of Dentistry, University of Oslo, Blindern, P.O. Box 1109, 0317 Oslo, Norway; 2 Nordic Institute of Dental Materials (NIOM), Oslo, Norway; 3 School of Dentistry, Institute of Clinical Sciences, College of Medical and Dental Sciences, University of Birmingham, Birmingham, UK

**Keywords:** Antibacterial compounds, Calcium silicate, Characterization, Root-end filling, Root repair

## Abstract

**Objectives:**

To investigate the effect of inclusion of silver nano-particles (SNP) or bioactive glass (BG) on the surface characteristics and bacterial adhesion of prototype tricalcium silicate (TCS)–based cements alongside two commercial cements, under different aging periods and exposure conditions.

**Materials and methods:**

A basic formulation of radio-opacified TCS without (TZ-base) and with additions of SNP (0.5, 1, or 2 mg/ml) or BG (10 or 20%) was used. Biodentine and intermediate restorative material (IRM) served as reference materials. Material disks were immersed in ultrapure water or fetal bovine serum (FBS) for 1, 7, or 28 days. Surface roughness (*n* = 3), microhardness (*n* = 9), and wettability (*n* = 6) were analyzed by standard procedures. Adhesion of *Enterococcus faecalis* was assessed by fluorescence microscopy (*n* = 5). Data from these assays were evaluated for normality and comparisons among groups were conducted with statistical procedures (*p* < 0.05 for significance).

**Results:**

The surface morphology of SNP- and BG-containing cements had higher roughness values than TZ-base after 28 days (*p* < 0.05). No differences in microhardness were observed among prototype cements (*p* > 0.05). Biodentine presented smooth surface characteristics and the highest hardness values (*p* < 0.05). The FBS-immersion resulted in surface reactions in prototype materials and Biodentine, depicted with scanning electron microscopy. All 1- and 7-day prototype cements showed negligible bacterial adhesion, while in Biodentine and IRM, noticeable *E. faecalis* adherence was observed from day 1 (*p* < 0.05).

**Conclusions:**

Incorporation of SNP or BG did not improve the antibacterial effect of the experimental cement; all 28-day aged materials failed to inhibit bacterial adherence. The measured physical parameters did not appear to be related to the degree of bacterial adhesion. Exposure of TCS-based cements in FBS resulted in surface reactions, which did not affect bacterial adhesion.

**Clinical relevance:**

Changes in the surface characteristics of prototype TCS-based cements by inclusion of SNP and BG or exposure to different environments did not affect bacterial adhesion. All experimental materials showed inferior physical properties and higher antibacterial effect than Biodentine.

**Supplementary Information:**

The online version contains supplementary material available at 10.1007/s00784-022-04655-y.

## Introduction

Root canal filling materials should provide a seal between the root canal system and the surrounding periodontal tissues [1]. The rationale is to inhibit bacterial penetration and consequent biofilm formation [2]. Materials used for apexification, perforation repair, or retrograde root filling face an additional challenge, as they have a larger contact area with underlying periodontal tissues compared to materials used for conventional root canal filling [1].

Tricalcium silicate (TCS)–based cements are materials with properties suitable for such procedures. Their main advantages are their hydraulic nature and the formation of calcium hydroxide upon hydration of the calcium silicate particles [3]. Release of calcium hydroxide may stimulate healing as well as provide an antimicrobial effect [4].

Mineral trioxide aggregate (MTA) is a Portland cement (PC)–based material developed specifically as a perforation repair material and for root-end filling [5]. Since its introduction in clinical dentistry, several materials based on pure TCS have been developed. PC was later replaced because of the potential of aluminum to leach to peripheral organs [6]. In addition, the new generations of hydraulic TCS-based cements contain additives aiming to enhance materials’ physico-chemical performance compared to MTA [7]. In Biodentine (Septodont, Saint Maur-des-Fosses, France), calcium carbonate and water-reducing agents are used for this purpose [8].

Antibacterial activity of endodontic materials may contribute to the eradication of bacteria that have survived the preceding disinfection procedures [9, 10]. Even in cases where root-end surgery has been performed and the area of infection has been removed, persistent bacteria may have the capacity to re-establish and cause a re-infection [11]. Ιn TCS-based cements, the antibacterial potential stems mainly from the high alkalinity due to hydroxyl ions of the calcium hydroxide by-product [12]. However, contact with blood may neutralize the antibacterial potential of MTA [13]. Overall, it seems that interactions of hydraulic materials with the environment can modulate their physico-chemical and biological properties [14, 15]. Calcium carbonate may be formed at the expense of calcium hydroxide [16], and the reduced alkalinity may limit the material’s antibacterial activity. Therefore, the surface properties as modified by interactions with the environment may play a role in the inhibition of bacterial adherence and consequent biofilm formation [17].

Taking into consideration the moderating effect of environmental conditions, introducing an antibacterial agent in TCS-based cements such as silver nano-particles (SNP) could be beneficial. SNP can penetrate the dentinal tubules [18] and limit bacterial growth by releasing free silver ions [19]. At the same time, as root-end filling and root-repair materials are usually placed in a field of chronic inflammation where tissue destruction prevails, the potential of a material to stimulate the post-treatment healing of the periodontal tissues is as important as the antibacterial effect. Inclusion of bioactive glass (BG) into TCS could contribute to the balance between these desirable properties [20]. A recent study showed that 10% addition of different types of BG to Biodentine resulted in marked apatite formation upon its surface suggesting an enhanced bioactivity potential [21]. The bioglass formulation BG 45S5 consists of silicon dioxide, calcium oxide, sodium oxide, and diphosphorus pentoxide and was the first material introduced in medicine that could induce osteogenesis and create a bond with the host bone tissue [22]. BG 45S5 also has a moderate, pH-dependent antibacterial effect [23].

Dentinal and material surfaces may serve as footholds for bacteria to attach and multiply with biofilm formation and produce disease [24]. Modifying the chemistry of biomaterials in order to limit biofilm formation or enhance their bioactivity would seem beneficial, provided the modifications do not negatively influence essential physical properties. The main aim of the current study was to investigate the surface characteristics as well as the bacterial adhesion of prototype TCS-based cements with or without incorporation of SNP or BG 45S5. The null hypothesis studied was that incorporation of SNP or BG 45S5 in hydraulic TCS-based cements will not have any effect on the cements’ surface characteristics nor in the inhibition of bacterial adhesion. An additional aim was to explore changes in the adhesion patterns in connection to surface characteristics across exposure to different environmental conditions and aging periods. For comparison, these properties were also investigated in two commercial materials, a TCS-based cement that contains modifications from the conventional synthesis of a radio-opacified TCS cement (Biodentine) and a zinc-oxide eugenol-based material (intermediate restorative material; Dentsply Sirona, Charlotte, NC, USA). Both are used in root-end filling or root repair procedures.

## Materials and methods

### Test materials

The following materials were used in the study:

- Tricalcium silicate cement (TCS; CAS No: 12168–85-3, American Elements, Los Angeles, CA, USA) with 20% weight-replacement of zirconium oxide (ZO; Koch-Light Laboratories, Colnbrook, Bucks, UK) (TZ-base).
- TZ-base with 10% or 20% replacement by weight of the TCS with bioactive glass 45S5 (BG; Cas No: 65997–17-3, 10 µm particle size, Mo-Sci Corporation, Rolla, MO, USA) (TZ-bg10, TZ-bg20 respectively).
- TZ-base with incorporation of 0.5 mg/ml, 1 mg/ml, or 2 mg/ml silver nano-particles (SNP; CAS No: 7440–22-4, < 100 nm particle size, Sigma-Aldrich, Gillingham, UK) (TZ-Ag0.5, TZ-Ag1, and TZ-Ag2 respectively), following dispersion of silver nano-powder in ultrapure water (water; Elix Essential 5 UV Water Purification System, Merck KGaA, Darmstadt, Germany).
- Biodentine (Septodont, Saint Maur-des-Fosses, France).
- Intermediate restorative material (IRM; Dentsply Sirona, Charlotte, NC, USA).

### Material preparation

#### Dispersion of silver nano-particles (SNP)

The NANOGENOTOX dispersion protocol was followed with slight modifications [25]. In brief, 12 mg SNP was pre-wet in 30 µl ethanol. Consequently, 5.97 ml water was slowly added in the solution resulting in a 2-mg/ml SNP concentration, instead of a 2.56-mg/ml concentration specified in the original protocol. The solution was then placed in an ice bath and sonicated (VCX 130, Sonics & Materials, Newtown, CT, USA). Further dilutions were prepared (1 mg/ml and 0.5 mg/ml). Sonication of SNP solution was carried out immediately before material mixing.

#### Mixing and placement

Prototype materials were hand-spatulated with water or the respective SNP solutions. The liquid/powder ratio employed was 0.35 ml/g. Commercial materials were handled according to the manufacturer’s instructions.

**Fig. 1:**
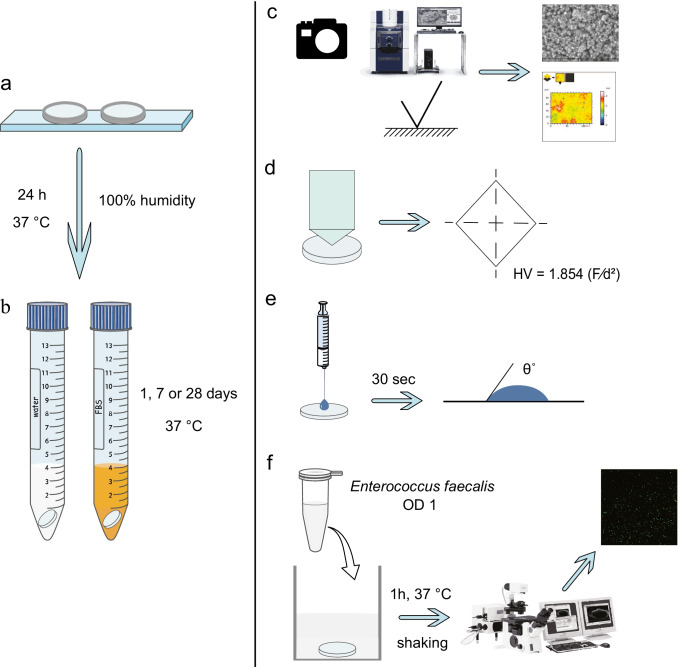
Schematic representation of the methodology. Test materials were compacted inside Teflon disks upon microscope slides (**a**). Consequently, they were immersed in 4 ml ultrapure water or fetal bovine serum (**b**). After the specified aging periods, specimens were subjected to photography, SEM, EDX, and surface roughness analysis (**c**) and assessment of surface microhardness (**d**) and wettability (**e**) as well as adhesion assay (**f**)

Materials were compacted inside Teflon disks (9 mm internal diameter, 1 ± 0.1 mm thickness) upon glass microscope slides (Fig. 1a). They were covered with a wet gauze and allowed to set for 24 h at 37 °C. After this period, the material specimens were immersed in 4 ml water or fetal bovine serum (FBS; F7524, Merck, Darmstadt, Germany). Samples were incubated for 1, 7, or 28 days at 37 °C (Fig. 1b). Consequently, they were retrieved, vacuum desiccated, and subjected to testing, except for the ones used in the adhesion assay, which were tested immediately after the respective aging periods.

The immersion medium was replaced with fresh one every 7 days.

### Material characterization

#### Evaluation of radio-opacity

Prototype materials (*n* = 3 per group) were tested in order to verify that the 20% ZO-incorporation induced adequate radio-opacity to the cements in compliance with the ISO 6876:2012 requirements [26]. Briefly, the material pellets and a 1-mm increment aluminum step wedge (1–10 mm thickness) were placed upon a photo-stimulable phosphor plate (VitaScan, Durr Dental, Bietigheim-Bissingen, Germany). Digital radiographs were acquired by a standard X-ray machine using an exposure time of 0.80 s at 10 mA, tube voltage at 65 ± 5 kV, and a cathode–target film distance of 30 cm. Radiographs were consequently processed and the digital images obtained served for the interpretation of results as described by Formosa et al. [27].

#### Photography

Specimens (*n* = 3) were photographed in a dark background to identify differences macroscopically in their color or structure after immersion in different media or from the incorporation of different components (Fig. 1c).

#### Scanning electron microscopy (SEM), energy dispersive X-ray (EDX) analysis, and surface roughness assessment

The materials (*n* = 3 per group) were placed upon carbon tapes and imaged with a scanning electron microscope (TM4000Plus II, Hitachi, Tokyo, Japan). Images were obtained by combining a backscattered electron and secondary electron signal detector (mix signal). Additionally, for the surface roughness analysis, 4 backscattered images were obtained at each observation field at 200 × magnification with a quad-type backscatter electron detector. Stereoscopic reconstruction in a 3D model of these images was performed with the use of a suitable software (MountainsMap; Digital Surf, Besançon, France). Eighteen surface roughness values (Ra) were consequently obtained from each 3D model following calibration of the program with an artificially induced consistent angulation upon the surface of a reference material. TZ-base was used for that purpose (Fig. 1c).

#### Surface microhardness assay

Materials (*n* = 9 per group) were subjected to Vicker’s microhardness testing (Duramin-40 A1, Struers, Rotherham, UK) (Fig. 1d). Values were obtained after exposing the samples to a 100-g load for 15 s of dwelling time. Three to five measurements were conducted per sample in non-overlapping planes. The Vickers hardness number (HV) was consequently automatically calculated from the program equipment using the following equation:

$$HV=1.854(F/{d}^{2})$$

#### Wettability assessment

Contact angle measurements (*n* = 6 per group) were taken using a contact angle goniometer (NRL 100–10, rame-hart, Mountain Lakes, NJ, USA). A micro-syringe was used to deliver a 10-µl drop of water upon the material surface. The angle was determined within 30 s of placement of the drop. Two measurements were conducted per sample (Fig. 1e).

### Assessment of bacterial adhesion

*Enterococcus faecalis* OG1RF, which expresses the green-fluorescent protein [28], was taken from frozen stock cultures and incubated overnight in Tryptic Soy Broth (TSB; Sigma-Aldrich, Gillingham, UK) at 37 °C with 5% CO_2_ in a humidified atmosphere. The following day, bacterial cultures were centrifuged and suspended in phosphate buffered saline (PBS; Fisher Scientific, Waltham, MA, USA) in order to prepare a bacterial inoculum with optical density (OD) 1.0 at 600 nm (approximately 1 × 10^8^ CFU/ml).

The material specimens (*n* = 5 per group) were placed inside 48 well-plates (Thermo Fisher Scientific, Waltham, MA, USA) and exposed to 700 µl of the *E. faecalis* inoculum for 1 h at 37 °C in a shaking incubator. Subsequently, they were carefully shaken and rinsed with sterile water to remove loosely attached bacteria. For positive control, sterile membrane filters (MF-Millipore, 0.45 µm pore size, Merck, Darmstadt, Germany) cut to the same diameter (9 mm) as the material specimens were used. Imaging of viable *E. faecalis* cells upon the material surface was performed with a confocal laser scanning microscope (CLSM; Olympus FluoView FV1200, Olympus, Tokyo, Japan) with a 60 × water immersion lens. Three images were obtained per specimen upon different areas of the material. Images (480 × 480 pixel size) were consequently processed with ImageJ (US National Institute of Health, Bethesda, MD, USA). Using the “find maxima” algorithm in the program and selecting a noise threshold of 12 towards a representative part of the image (1/4 of the initial size-240 × 240 pixel size), bacterial cells were depicted and automatically counted (Fig. 1f).

### Statistical analyses

All quantitative results were analyzed statistically, except for the radio-opacity values which were assessed qualitatively in terms of if the ISO standards [26] were fulfilled. IBM SPSS Statistics software version 27 (IBM, Armonk, NY, USA) was employed. Each set of results was assessed for normality according to the Shapiro–Wilk test. The majority of contact angle values did not follow the normal distribution and were thus analyzed with non-parametric Kruskal–Wallis test adjusted by the Bonferroni correction or Mann–Whitney *U* test. For groups with only zero values, a one sample Wilcoxon rank-sum test was used for comparisons with other groups. All other data were analyzed with ANOVA and Bonferroni post-hoc tests for multiple comparisons. Multiple comparisons for data for surface roughness, microhardness, and adhesion assays were performed among different materials for similar aging period and immersion medium or for the same materials along different aging periods and immersion medium. The significance level to all analyses was set at *p* = 0.05.

## Results

### Characterization

#### Radio-opacity

All prototype materials had adequate radio-opacity according to ISO 6876 [26] (Table 1). Further experiments for prototype cements were therefore conducted with the 20% ZO-replacement.

**Table 1:** Mean and standard deviation of radio-opacity (mm aluminum) of prototype cements

Material	Radio-opacity (mm aluminum)
TZ-base	3.6 (0.5)
TZ-bg10	3.5 (0.3)
TZ-bg20	3.4 (0.5)
TZ-Ag0.5	3.7 (0.8)
TZ-Ag1	3.6 (0.4)
TZ-Ag2	3.4 (0.2)

#### Macroscopic assessment of material surface

**Fig. 2:**
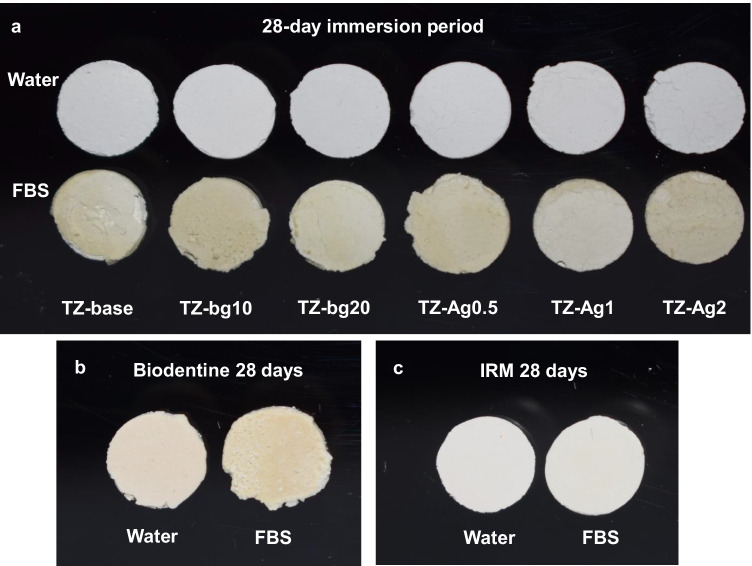
Indicative images of prototype materials (**a**), Biodentine (**b**), and IRM (**c**) after a 28-day immersion period in ultrapure water (water) or fetal bovine serum (FBS)

The color of prototype materials was not visibly affected by the incorporation of SNP (Fig. 2a). After the immersion periods in FBS, prototype cements experienced a visible yellow discolouration (Fig. 2a), while changes in Biodentine surface were evident as white depositions particularly after 28 days (Fig. 2b). No change in the color or structure of IRM was evident macroscopically (Fig. 2c).

#### SEM, EDX, and surface roughness

**Fig. 3:**
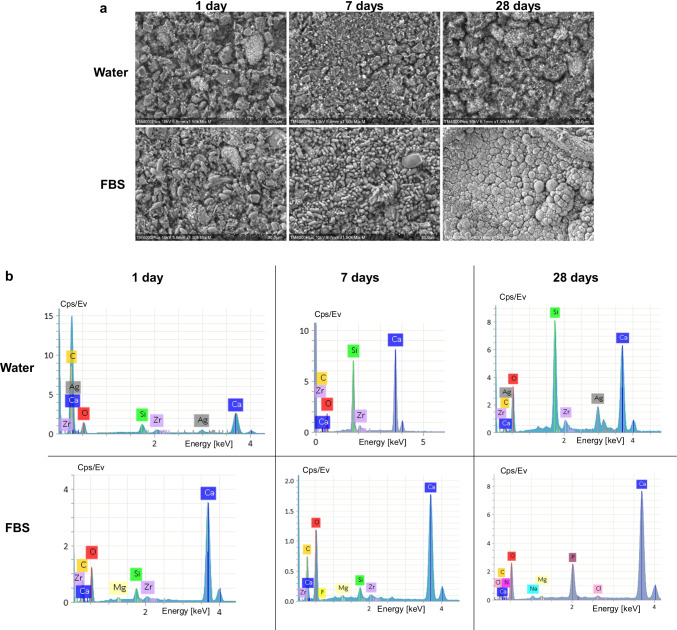
Representative scanning electron micrographs of TZ-Ag2 acquired with a mix of back-scatter and secondary electron signal detectors (1500 × magnification) after exposure to ultrapure water (water) or fetal bovine serum (FBS) for 1, 7, or 28 days (**a**). Representative energy-dispersive spectroscopic scans of selected spectrums (**b**)

SEM images of prototype cements after immersion in water showed similar surface morphology. The material matrix appeared gradually more homogenized after 7 and 28 days (Fig. 3a, Online Resource 1). The main elements in the EDX scans were calcium, silicon, oxygen, and zirconium, while phosphate and sodium was also evident in the BG-containing materials. Silver was rarely depicted in the SNP-containing cements, mainly in the TZ-Ag2 (Fig. 3b).

For surface roughness assessments in the water-immersed materials (Table 2), all BG- and SNP-containing cements reported higher Ra values than the rest after 28 days (*p* < 0.01), with TZ-bg20 having the highest Ra (*p* < 0.001). The Ra values of TZ-base and TZ-Ag1 showed a significant decrease between 7 and 28 days (*p* < 0.05), while those of TZ-bg20 increased between 1 and 28 (*p* < 0.05). Biodentine had a smoother surface morphology than all the prototype cements in all aging periods (*p* < 0.001), without fluctuations in Ra (*p* > 0.05).

**Table 2:** Mean and standard deviation of surface roughness Ra (µm) for materials after immersion in water or FBS for 1, 7, or 28 days. Read horizontally, the same small superscript letter indicates no statistically significant differences between different aging periods and immersion solutions within the same material (*p* > 0.05). Read vertically, the same capital letter shows non-statistically significant difference among materials for the exact same conditions of testing (aging period and immersion medium) (*p* > 0.05). In 28-day water-immersed samples, all BG- and SNP-containing cements reported higher Ra than the rest materials, with TZ-bg20 having the highest values (*p* < 0.05). Water-immersed Biodentine and IRM had a smoother surface morphology than all the prototype cements in all aging periods (*p* < 0.05). The 28-day FBS-immersed prototype cements had higher Ra than the respective water-exposed samples (*p* < 0.05), except for TZ-Ag2 (*p* > 0.05)

Surface roughness (Ra)						
Material	1 day		7 days		28 days	
	Water	FBS	Water	FBS	Water	FBS
**TZ-base**	0.095 (0.004)^Α.Β−a.b^	0.115 (0.006)^A.B−a^	0.09 (0.002)^A.B−b^	0.112 (0.008)^A−a^	0.063 (0.001)^Α−c^	0.113 (0.022)^A.B−a.b^
**TZ-bg10**	0.083 (0.005)^Α−a^	0.117 (0.016)^A.B−a.b^	0.104 (0.001)^A−a^	0.115 (0.008)^A−a^	0.083 (0.002)^Β−a^	0.171 (0.056)^A−b^
**TZ-bg20**	0.091 (0.006)^Α−a^	0.099 (0.006)^A.C−a.b^	0.1 (0.005)^A.B−a.b^	0.125 (0.004)^A−c.d^	0.109 (0.008)^C−b.c^	0.127 (0.004)^A.B−d^
**TZ-Ag0.5**	0.096 (0.001)^Α.Β−a^	0.109 (0.007)^A.B−a^	0.1 (0.004)^A.B−a^	0.125 (0.001)^A−a^	0.08 (0.001)^B−a^	0.211 (0.051)^A−b^
**TZ-Ag1**	0.094 (0.0001)^Α.Β−a.b^	0.123 (0.007)^B−c.d^	0.104 (0.004)^A−a.d^	0.125 (0.008)^A−c.d^	0.082 (0.005)^B−b^	0.134 (0.012)^A.B−c^
**TZ-Ag2**	0.106 (0.011)^Β−a.b^	0.113 (0.001)^A.B−a.b^	0.087 (0.006)^B−a.b^	0.12 (0.002)^A−a.b^	0.083 (0.002)^B−b^	0.119 (0.026)^A.B−a.b^
**Biodentine**	0.038 (0.003)^C−a^	0.079 (0.01)^C.D−a.b^	0.044 (0.005)^C−a^	0.12 (0.024)^A−a.b^	0.038 (0.001)^D−a^	0.133 (0.061)^Α.B−b^
**IRM**	0.036 (0.003)^C−a^	0.039 (0.003)^D−a^	0.041 (0.007)^C−a^	0.044 (0.01)^B−a^	0.03 (0.001)^D−a^	0.042 (0.01)^B−a^

**Fig. 4:**
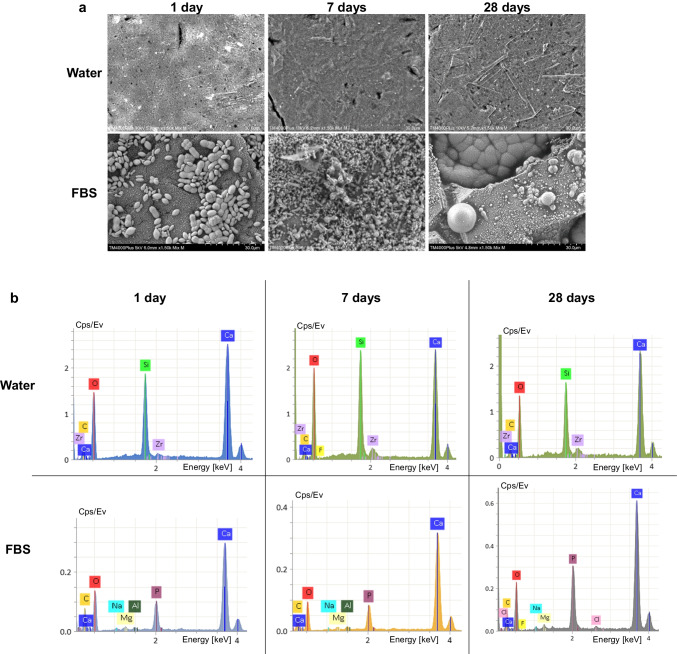
Scanning electron micrographs of Biodentine acquired with a mix of back-scatter and secondary electron signal detectors (1500 × magnification) after exposure to ultrapure water (water) or fetal bovine serum (FBS) (1500 × magnification) (**a**). Representative energy-dispersive spectroscopic scans of selected spectrums (**b**)

Immersion in FBS altered completely the surface characteristics of TCS-based cements (prototype cements and Biodentine). SEM images revealed the precipitation of organic compounds and consequent reaction with the cements’ surface, which was gradually more evident for longer immersion period (Figs. 3a and 4a; Online Resources 1 and 2). The EDX analysis showed that the particles that were formed consisted mainly of calcium and oxygen as well as carbon or phosphorus. Traces of sodium, magnesium, nitrogen, and chlorine were also observed occasionally in relatively low amounts (Figs. 3b and 4b, Online Resource 1). Consequently, a rougher surface morphology was observed in all cases for TCS-based cements upon immersion in FBS compared to the respective water ones for the same aging periods, which was statistically significant for 28 days samples (*p* < 0.05), except for TZ-Ag2 (*p* > 0.05). Biodentine did not report any significant difference from the prototype cements after 7 and 28 days (*p* > 0.05) (Table 2).

**Fig. 5:**
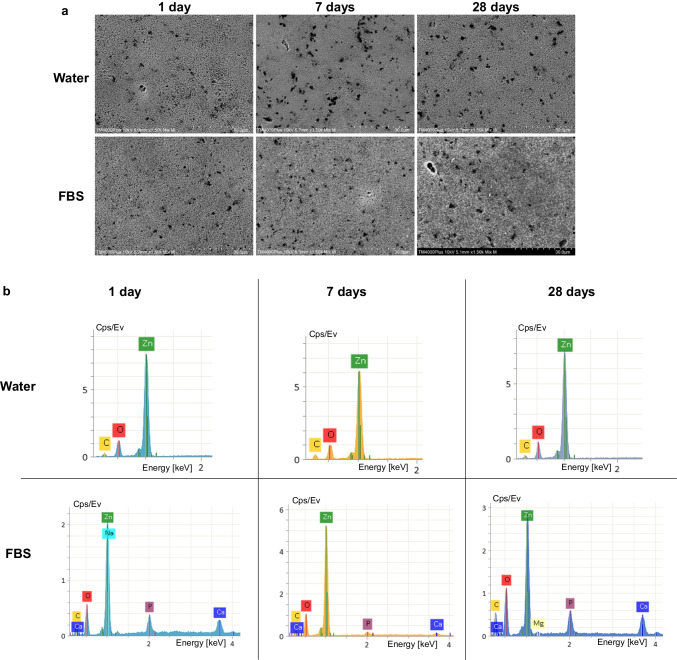
Representative scanning electron microgaphs of IRM acquired with a mix of back-scatter and secondary electron signal detectors (1500 × magnification) after exposure to ultrapure water (water) or fetal bovine serum (FBS) (1500 × magnification) for 1, 7, or 28 days (**a**). Representative energy-dispersive spectroscopic scans of selected spectrums (**b**)

IRM showed no alterations in Ra values for the different aging periods or immersion media (*p* > 0.05) (Fig. 5a). The Ra of IRM was significantly lower than all other materials after immersion in water (*p* < 0.001), except compared with Biodentine (*p* > 0.05). EDX analysis showed the presence of calcium, phosphorus, and occasionally sodium and magnesium in the FBS-immersed IRM samples in addition to zinc and oxygen that were depicted in the water-immersed ones (Fig. 5b).

#### Surface microhardness

Prototype materials had overall significantly lower microhardness values than Biodentine for all test conditions (*p* < 0.001), as well as IRM (*p* < 0.01) except for the 28-day water-immersed samples (*p* > 0.05) (Table 3). Incorporation of BG or SNP did not alter the microhardness of the prototype cements for any aging period for both immersion media (*p* > 0.05).

**Table 3:** Mean and standard deviation of surface microhardness of test materials after 1, 7, or 28 days immersion in water or FBS. Read horizontally, the same small superscript letter indicates no statistically significant differences between different aging periods and immersion solutions within the same material (*p* > 0.05). Read vertically, the same capital letter shows non-statistically significant difference among materials for the exact same conditions of testing (aging period and immersion solution) (*p* > 0.05). Prototype cements’ microhardness was not affected by SNP- or BG-incorporation (*p* > 0.05). Biodentine had the highest values in all test conditions, while the microhardness of 28-day FBS-immersed Biodentine was higher than the respective water-exposed (*p* < 0.05)

Surface microhardness (HV)						
Material	1 day		7 days		28 days	
	Water	FBS	Water	FBS	Water	FBS
**TZ-base**	8.05 (1.79)^A−a,b^	7.08 (1.79)^A−a^	10.59 (2.23)^A−b^	6.34 (1.13)^A−a^	8.25 (0.87)^A−a,b^	8.75 (2.38)^A−a,b^
**TZ-bg10**	7.64 (1.18)^A−a^	7.18 (1.08)^A−a^	7.83 (1.47)^A−a^	6.85 (1.83)^A−a^	8.1 (1.26)^A−a^	8.6 (2.4)^A−a^
**TZ-bg20**	6.23 (1.38)^A−a,b^	6.77 (1.41)^A−a,b^	8.07 (1.42)^A−a^	5.47 (1.25)^A−b^	7.63 (1.59)^A−a,b^	8.5 (2.63)^A−a^
**TZ-Ag0.5**	7.11 (2.22)^A−a^	6.59 (1.3)^A−a^	9.74 (1.96)^A−b^	7.31 (2.23)^A−a,b^	8.26 (1.45)^A−a,b^	7.99 (1.4)^A−a,b^
**TZ-Ag1**	7.46 (1.72)^A−a^	7.06 (1.37)^A−a^	8.98 (1.96)^A−a^	7.38 (2.02)^A−a^	8.3 (1.43)^A−a^	8.67 (2.43)^A−a^
**TZ-Ag2**	6.55 (1.3)^A−a^	7.56 (1.62)^A−a,b^	10.28 (1.32)^A−c^	7.54 (1.39)^A−a,b^	9.98 (2.74)^A−b,c^	8.96 (1.59)^A−a,c^
**Biodentine**	55.23 (6.87)^B−a,b^	53.8 (11.77)^B−a,b^	49.51 (2.6)^B−a,c^	47.88 (2.74)^B−a,c^	35.53 (4.89)^B−c^	67.56 (22.31)^B−b^
**IRM**	19.14 (3.2)^C−a,b^	23.3 (4.24)^C−a,d^	14.3 (2.03)^C−b,c^	28.51 (3.31)^C−d^	8.23 (1.05)^A−c^	44.21 (8.74)^C−e^

In the water-immersed materials, Biodentine reported a significant decrease in hardness values between the 1- and 28-day samples (*p* < 0.01), while it increased in FBS from the 7- to 28-day period (*p* < 0.01). Hardness of Biodentine in FBS was significantly higher compared to the water-immersed samples after the 28-day immersion period (*p* < 0.001).

IRM had significantly lower microhardness values than Biodentine during all tested periods (*p* < 0.001). Overall, IRM had a similar pattern as Biodentine in terms of fluctuations in hardness values, reporting a significant decrease or increase in the 28-day water and FBS samples, respectively (*p* < 0.001 for comparisons between 1- and 28-day water-immersed samples and for comparisons between 1- or 7-day FBS-immersed samples with the 28-day ones)*.* Additionally, the 7- and 28-day FBS samples reported higher values than the respective water ones (*p* < 0.001).

#### Contact angle

Complete wetting was observed in all the water-immersed prototype cements (Table 4). In the FBS-immersed prototype materials, the hydrophilicity was moderated for 1-day samples (*p* < 0.05) but it was gradually re-established in the 7-day samples until complete wetting was reported again after 28 days. Biodentine had a similar wettability pattern as the prototype cements, without however reporting complete wetting at any evaluation period. FBS immersion did not alter Biodentine’s wettability significantly in any period (*p* > 0.05). IRM had a different behavior, as the immersion in FBS resulted in relatively higher wettability, which was significantly different from the water-immersed samples after 7 days (*p* < 0.001). No changes occurred throughout the immersion periods for both water- and FBS-immersed samples of IRM (*p* > 0.05).

**Table 4:** Median and interquartile range of contact angle measurements of test materials after 1, 7, or 28 days immersion in water or FBS. Read horizontally, same small superscript letters indicate no statistically significant differences between different aging periods and immersion solutions within the same material (*p* > 0.05). Read vertically, same capital letters show non-statistically significant differences among materials for the exact same conditions of testing (aging period and immersion solution) (*p* > 0.05). FBS-immersed prototype materials showed lower hydrophilicity than the respective water samples, particularly after 1 day (*p* < 0.05), which was, however, gradually re-established through time

Contact angle values (°)						
Material	1 day		7 days		28 days	
	Water	FBS	Water	FBS	Water	FBS
**TZ-base**	0^A−a^	77 (13)^A,B−b^	0^A−a^	0 (82)^A−a,b^	0^A−a^	0^A−a^
**TZ-bg10**	0^A−a^	91 (27)^B−b^	0^A−a^	46 (65)^A−a^	0^A−a^	0^A−a^
**TZ-bg20**	0^A−a^	66 (16)^A,B−b^	0^A−a^	0 (27)^A−a,b^	0^A−a^	0 (63)^A,B−a,b^
**TZ-Ag0.5**	0^A−a^	82 (47)^A,B−b^	0^A−a^	0 (43)^A−a^	0^A−a^	0^A−a^
**TZ-Ag1**	0^A−a^	63 (27)^A,B−b^	0^A−a^	24 (57)^A−a^	0^A−a^	0^A−a^
**TZ-Ag2**	0^A−b^	80 (48)^A,B−b^	0^A−a^	34 (89)^A−a,b^	0^A−a^	0^A−a^
**Biodentine**	45 (26)^B−a^	70 (30)^A.B−a^	38 (63)^B−a^	90 (26)^A−a^	45 (45)^B−a^	38 (59)^B−a^
**IRM**	88 (25)^C−a,b^	40 (25)^A−a,b^	90 (20)^C−a^	35 (11)^A−b^	81 (14)^C−a^	37 (35)^B−a,b^

### Bacterial adhesion assay

**Fig. 6:**
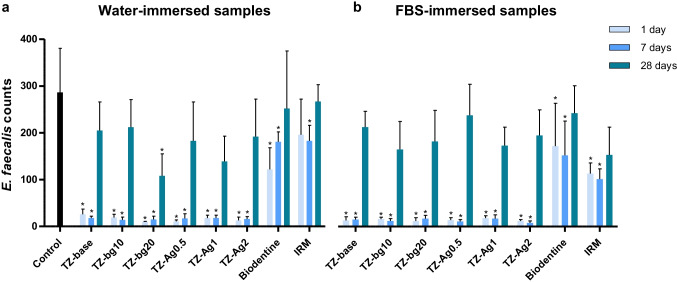
Mean and standard deviation of bacterial adhesion upon the surface of materials after a 1-h agitation period at 37 °C in an *Enterococcus faecalis* inoculum. Materials were previously aged in ultrapure water (water) (**a**) or fetal bovine serum (FBS) (**b**) for 1, 7, or 28 days. Control corresponds to sterile membrane filters cut to the same diameter (9 mm) as the material specimens. Asterisk indicates statistically significant difference from the control (*p* < 0.05)

Prototype materials showed an overall low amount of *E. faecalis* adhesion after 1 and 7 days of aging, regardless of the immersion liquid. No significant differences were reported among them for these periods (*p* > 0.05) (Fig. 6, Online Resource 3a). Additionally, significantly fewer bacteria were observed on the surface of the prototype cements in comparison to Biodentine (Online Resource 3b) and IRM for the 1- and 7-day conditions both for water- and FBS-immersed samples (*p* < 0.001). In the 28-day samples, a significant increased adhesion of the prototype cements was observed for both immersion media (*p* < 0.001).

The TZ-bg20 water-immersed 28-day samples had the lowest bacterial adhesion, which was only significantly different than the positive control (*p* < 0.05). No other differences were observed among prototype materials, Biodentine, and IRM for both the water- and FBS-immersed samples after 28 days (*p* > 0.05).

In Biodentine, all samples allowed *E. faecalis* adhesion. However, bacterial adhesion was significantly lower than the positive control for the 1- and 7-day samples (*p* < 0.05). No significant differences were reported between media for the same evaluation periods (*p* > 0.05).

Bacterial adhesion was also observed in IRM, while after 28 days of aging, the water-immersed samples had significantly more bacteria compared to the FBS ones (*p* < 0.05).

## Discussion

The present study assessed whether the incorporation of silver nano-particles or bioactive glass in TCS-based cements with rationale of use for root repair or root-end filling procedures could induce significant alterations in their surface characteristics and consequently modify their bacterial adhesion profile. The effect of immersion in a protein-rich solution was also investigated. Our results indicated that the additions caused an increase in surface roughness; however, this was not accompanied by changes in the adhesion patterns. The null hypothesis was therefore partially rejected. It was also shown that surface characteristics of TCS-based materials were significantly altered upon exposure to a protein-rich environment, again without affecting the bacterial adhesion patterns. Biodentine’s better physical properties and smoother surface characteristics in comparison to the non-modified prototype cement were accompanied by a negligible early inhibition of bacterial adhesion.

Incorporation of BG in obturation materials has been reported to result in better adaptation to the surrounding dentin walls, probably due to the ionic exchange taking place at the interface and expansion of BG particles upon hydration [29]. BG is dissolved in contact with water leading to leaching of calcium and phosphate ions and formation of an intermediate layer rich in silicon oxide [30]. Leaching plays a significant role in the induction of healing processes [31]. A pronounced apatite-forming ability has been previously reported following BG-additions in Biodentine [21]. An antibacterial effect is also evident due to the increase in pH [23] and osmotic pressure [32]. These properties would seem beneficial in a root-repair material, and we therefore wanted to evaluate the surface modifications upon incorporation of BG compounds to TCS-based cements. We used BG 45S5 micro-particles as they appear superior in terms of their chemical profile in comparison to other BG types [33] and have similar characteristics to BG particles that have been previously incorporated in a commercial endodontic sealer [34, 35].

Similarly, silver nano-particles were considered candidates for improvement of the antibacterial properties of cements. Their antibacterial activity is attributed to the release of free silver ions that interact with sulfhydryl groups and nitrogen atoms in proteins and nucleic acids of bacterial cells and induce significant disruptions to their functionality [36]. At the same time, their nano-size enables them to penetrate into target cells and to cause cell damage by their oxidization potential [37]. Despite the well-researched potential of enhancement of the antibacterial activity of SNP-incorporation in various dental materials, such as resins [38], adhesives [39], endodontic medicaments [40], and irrigants [41, 42], there is still a controversy in the literature whether their desirable properties are derived from leaching of SNP to the immediate environment or through direct contact upon the material [43]. Furthermore, establishing an optimum concentration of silver nanoparticles for adequate antibacterial activity is challenging, as their reactivity depends on parameters that vary among the recruited compounds in the literature, namely, size, capping, and charge [44].

A commercial TCS-based cement was included in the study for comparison with the experimental formulations. Biodentine was selected due to its chemical similarities to the prototype cements, as it consists mainly of TCS cement and has ZO as radio-opacifier, in contrast to PC-based MTA. Additionally, it contains compounds that enhance its physico-chemical profile. Calcium chloride and a water-soluble polymer are used in the liquid to reduce setting time and improve physical properties, respectively. Calcium carbonate is also added to the cement to control the hydration reaction [45]. IRM was included in the study in order to investigate the effect of environmental conditions and aging period on a material of completely different chemical composition from the tested hydraulic cements, but similar clinical application. IRM is used for root-end filling procedures and has reported similar clinical success to MTA [46, 47].

Root-repair and root-end filling materials are placed in a challenging biological environment, where they interact with tissue fluids, blood, and residual bacteria that might have survived the attempts of disinfection [48]. The materials can therefore be crucial in preventing bacteria from getting access to nutrients that can enable them to proliferate and cause a re-infection. The role of their morphological characteristics in this dynamic environment defines their antibacterial profile to a significant extent together with the potentially leachable components [49]. Only few studies have assessed antimicrobial properties of hydraulic cements from the perspective of their use for root-end filling or perforation repair procedures [50]. At the same time, introducing chemical compounds to the formulation of TCS cements for enhancement of antibacterial or biological properties should not compromise any of their existing physical properties.

A thorough characterization of materials’ surface characteristics was performed after aging in different environmental conditions and time periods. Exposing the materials to a protein-rich medium (FBS) sought to resemble the interactions that take place between materials and host tissue fluids [51]. The results may be compared with results obtained under the more controlled in vitro environment in the absence of any organic or inorganic compounds. Little research has been conducted on the antibacterial properties after contact with clinically relevant fluids, but one previous study indicated a reduction in activity of MTA following contact with blood and heparin [13]. To the best of our knowledge, this is the first study to evaluate antibacterial properties of Biodentine following exposure to a serum-containing environment. Testing was performed after three different incubation periods to follow changes in material properties as the hydration of cements was progressing until it was largely completed [3].

In the adhesion assay, direct bactericidal activity as well as anti-adherence surface characteristics may co-exist. Including longer incubation periods in our investigation enabled us to render these two roles more discrete, as the bactericidal effect might be decreased through time. The fluorescence assay for adhesion was compared in preliminary experiments with cultural data and found to yield similar, reliable, and reproducible results (data not shown). While the use of *E. faecalis* may not reflect fully the clinical scenario since endodontic infection has a polymicrobial etiology [52], it has been commonly used for in vitro and ex vivo evaluations of the antibacterial effect of root-end filling materials [50], and the results with a single organism are easily quantifiable and reproducible. The selection of the specific strain of *E. faecalis* that expresses the green-fluorescent protein enabled us to avoid excessive sample manipulation and any potential stain interference in the imaging process. Further investigations under a biofilm model might be useful as they could provide information on materials’ antibacterial potential under more extreme testing conditions.

Inclusion of BG altered the roughness profile of the cement along the hydration process. Even though all prototype cements that contained additives showed rougher surfaces at 28 days following water immersion comparing to the non-modified cement, the TZ-bg20 was the only material that increased in surface roughness eventually from day 1. This may be partially explained by the different hydration mechanisms for TCS and BG particles [3, 22]. A recent study showed that 20% incorporation of a different BG-type (Biosilicate) into prototype TCS achieved complete killing of planktonic *E. faecalis* in contrast to the non-modified cement [53]. Despite differences in the experimental design and the category of BG, the above findings are in line with ours: the 1-day prototype materials did not allow initial bacterial adhesion, possibly due to direct killing, while in the 28-day samples, TZ-bg20 had the lowest *E. faecalis* adhesion, which was significantly different from the positive control.

Silver nano-particles increased the roughness profile of cements after 28 days but did not have any other effect on the properties studied. Even though SNP have been used previously to improve the antibacterial properties of PC-based materials with reported success, the effect was only investigated 2 days after setting [54]. We found no change in the bacterial adhesion pattern from the addition of SNP. SNP might demand a longer interaction period with the negatively charged bacterial cells to achieve bacterial killing [55] following the early stages of bacterial adhesion, and it might also result from loss of SNP from the surface to the medium, rendering the surfaces relatively unaltered.

Biodentine showed an overall smoother surface morphology particularly up to 7 days after immersion, a result of the inclusion of the water-soluble polymer reducing cement flocculation [56]. In addition, decrease of the water/powder ratio in Biodentine increases the material hardness [45, 57]. This was evidenced by comparisons with the non-modified cement in our results. Bacteria adhered in large numbers to Biodentine. The material is moderately active towards *E. faecalis* [58]. Differences in the antibacterial activity in comparison with prototype cements might reflect a diverse extent of solubility and therefore leaching. Additionally, exposure of the materials to lower pH environments such as the bacterial inoculum could have resulted in a greater dissolution effect in the prototype cements, in contrast to Biodentine, which can withstand acidic environments [59].

Interestingly, the microhardness of Biodentine was reduced after long-term incubation in water, possibly due to continued dissolution. Changing of the immersion medium weekly might have affected microhardness, as it tends to increase leaching by not allowing buffering of the solution. In prototype materials and particularly in TZ-base, some minor fluctuations in the hardness values particularly between 7 and 28 days might have a similar explanation.

Immersion in FBS altered significantly the surface properties of all TCS-based materials. However, their behavior in the adhesion assay was unaltered. Material surfaces were progressively covered by products of the reaction of the calcium hydroxide with serum components, with SEM images and EDX analysis indicating gradual formation of a layer of calcium phosphate and mainly calcium carbonate. This layer decreased the hydrophilicity to some extent, particularly in the short-term immersion periods, while it resulted in reinforcement of surface hardness of Biodentine in the long term in contrast to its behavior in water. Alterations in the hydration process upon exposure to serum-containing environments have been described before both in vitro [13, 60] and in clinical conditions [16].

In IRM, even though the surface morphology of the material was not altered after the various exposure periods in FBS, the EDX analysis showed a qualitative modification with deposition of elements from the serum. Surface microhardness was consequently increased in FBS. This could be a result of a shift in the equilibrium of dissolution and uptake possibly caused by the serum components. Interestingly, IRM immersed in FBS showed higher wettability in combination with a reduction in the adhesion of *E. faecalis*. As the surface roughness of IRM remained stable in both media, the chemical changes in the material surface would seem responsible for the reduced bacterial adhesion. Increased hydrophilicity has been linked with reduced biofilm formation on dental resins [61] and may warrant further studies of hydrophilicity also of materials used for endodontic surgical procedures.

## Conclusions

Inclusion of silver nano-particles or bioactive glass did not affect the adhesion of *E. faecalis* in comparison to the non-modified composition. Exposure to FBS caused surface reactions in TCS-based cements that altered significantly their surface characteristics but did not affect the adhesion pattern. The measured physical parameters did not appear to be related to the degree of bacterial adhesion. Further studies should focus on the effect of leaching properties to the antibacterial profile upon alterations in the cement composition under different aging conditions.

## Supplementary Information

Below is the link to the electronic supplementary material.

Supplementary file1 (PPTX 3070 KB)

Supplementary file2 (PPTX 4.73 MB)

Supplementary file3 (PPTX 272 KB)
