# Supplementary material for: Surface characteristics and bacterial adhesion of endodontic cements
Source: Clin Oral Investig. 2022 Aug 5;26(12):6995–7009. doi: 10.1007/s00784-022-04655-y (PMC9708781; doi:10.1007/s00784-022-04655-y)
Supplement: Supplementary file 1 — Supplementary file1 (PPTX 3070 KB) [file 784_2022_4655_MOESM1_ESM.pptx]

## Slide 1
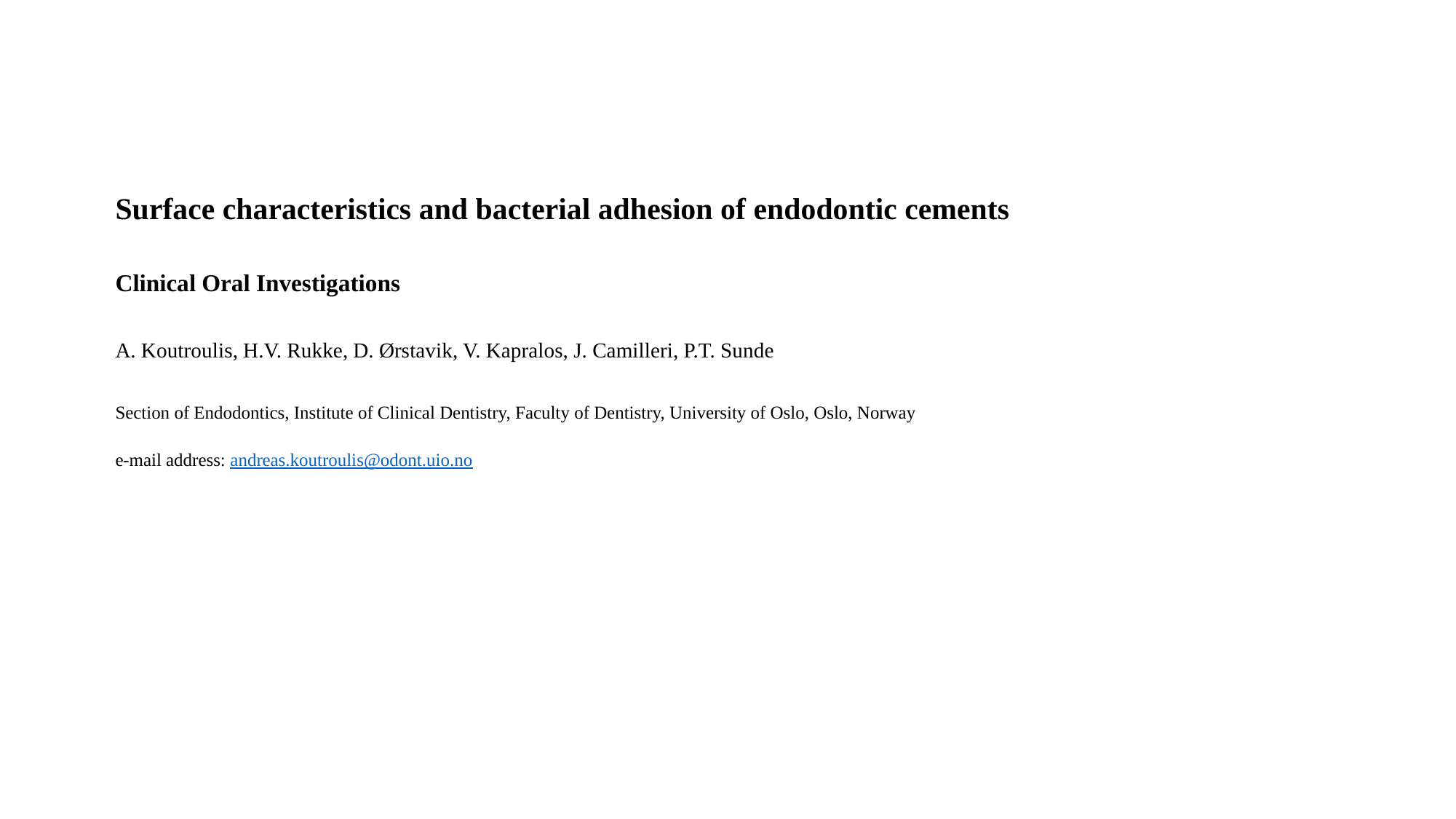

Surface characteristics and bacterial adhesion of endodontic cements
Clinical Oral Investigations
A. Koutroulis, H.V. Rukke, D. Ørstavik, V. Kapralos, J. Camilleri, P.T. Sunde
Section of Endodontics, Institute of Clinical Dentistry, Faculty of Dentistry, University of Oslo, Oslo, Norway
e-mail address: andreas.koutroulis@odont.uio.no

## Slide 2
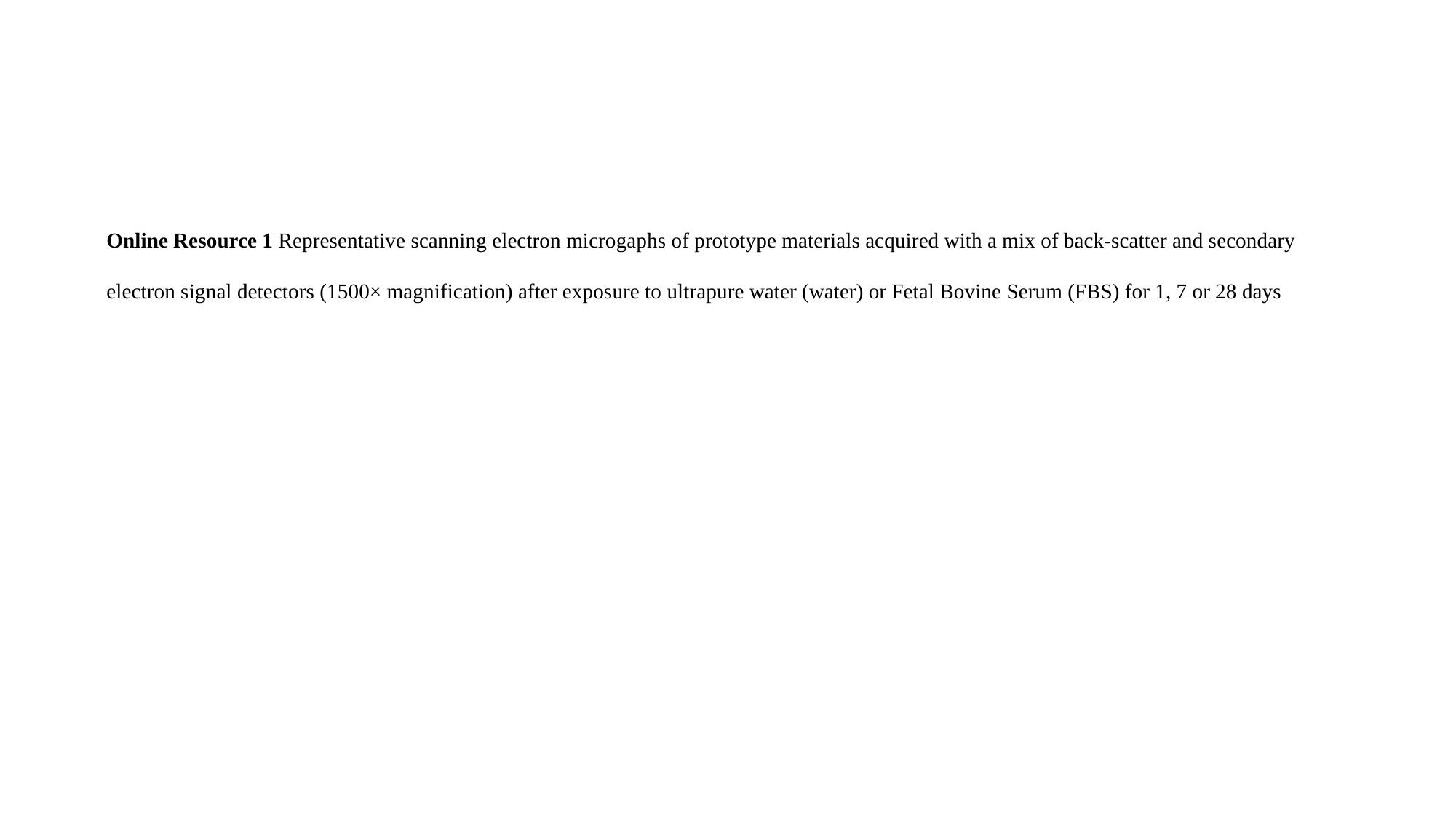

# Online Resource 1 Representative scanning electron microgaphs of prototype materials acquired with a mix of back-scatter and secondary electron signal detectors (1500× magnification) after exposure to ultrapure water (water) or Fetal Bovine Serum (FBS) for 1, 7 or 28 days

## Slide 3
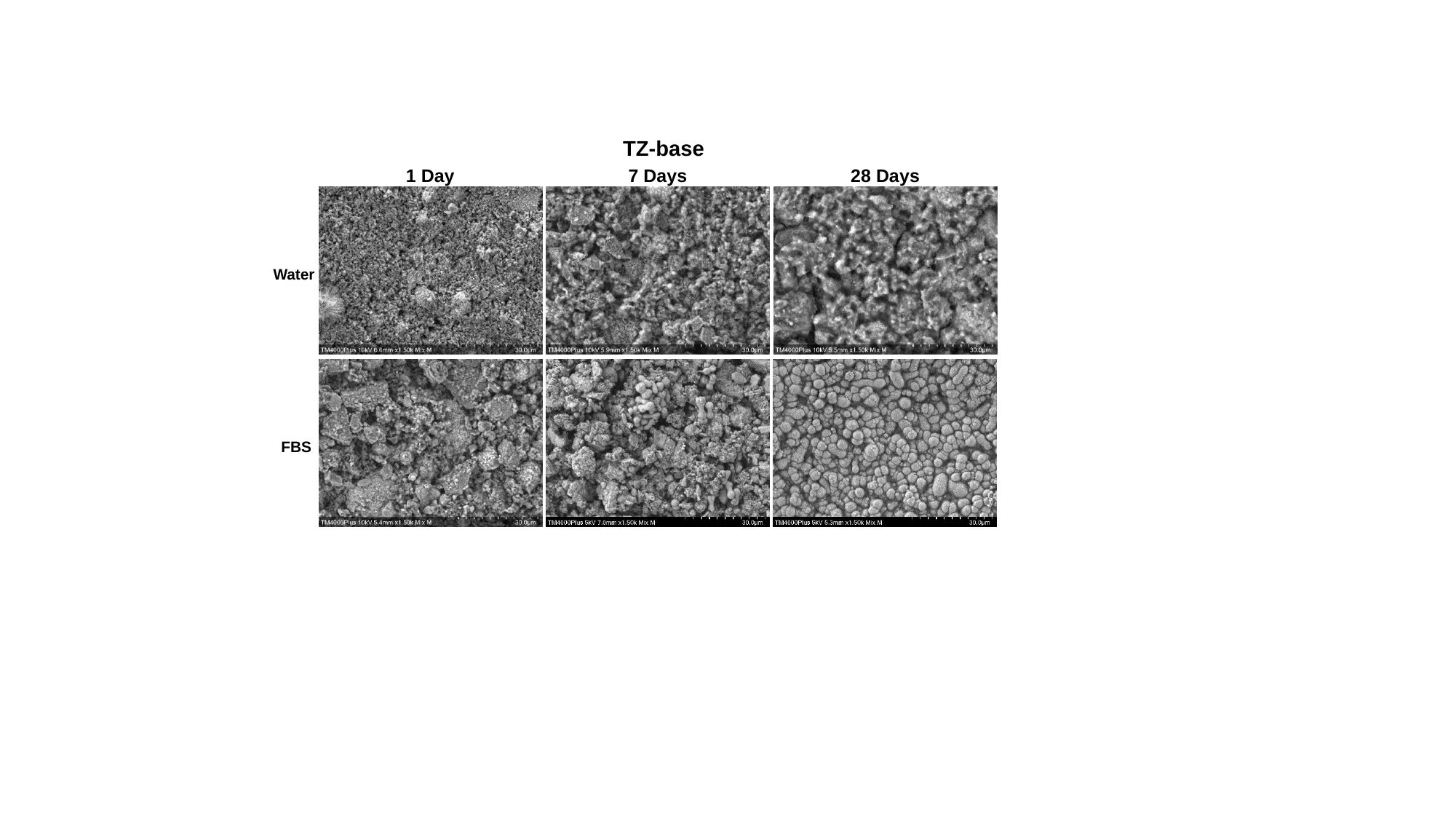

TZ-base
7 Days
28 Days
1 Day
Water
FBS

## Slide 4
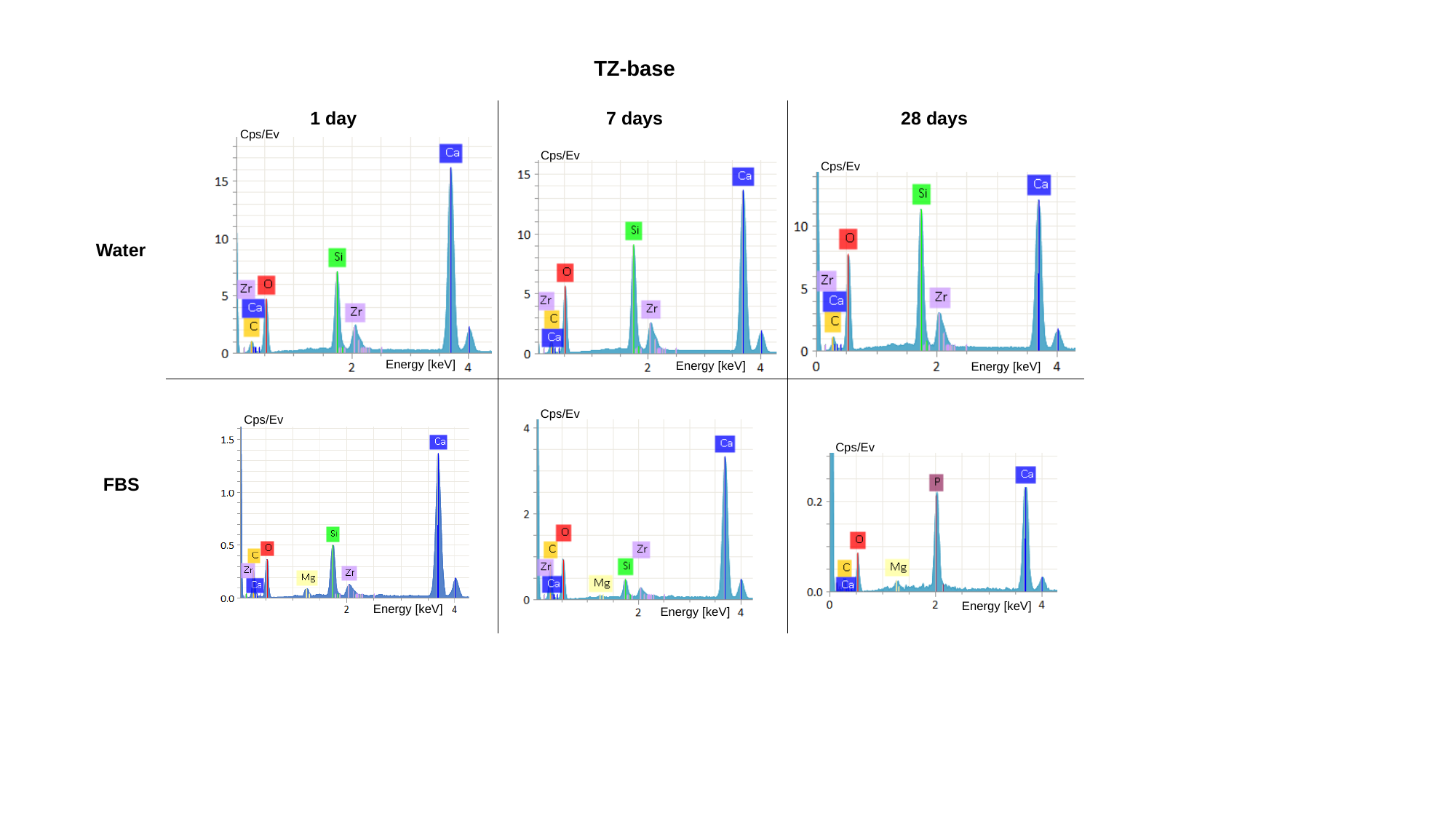

TZ-base
1 day
7 days
28 days
Cps/Ev
Energy [keV]
Cps/Ev
Energy [keV]
Cps/Ev
Energy [keV]
Water
Cps/Ev
Energy [keV]
Cps/Ev
Energy [keV]
Cps/Ev
Energy [keV]
FBS

## Slide 5
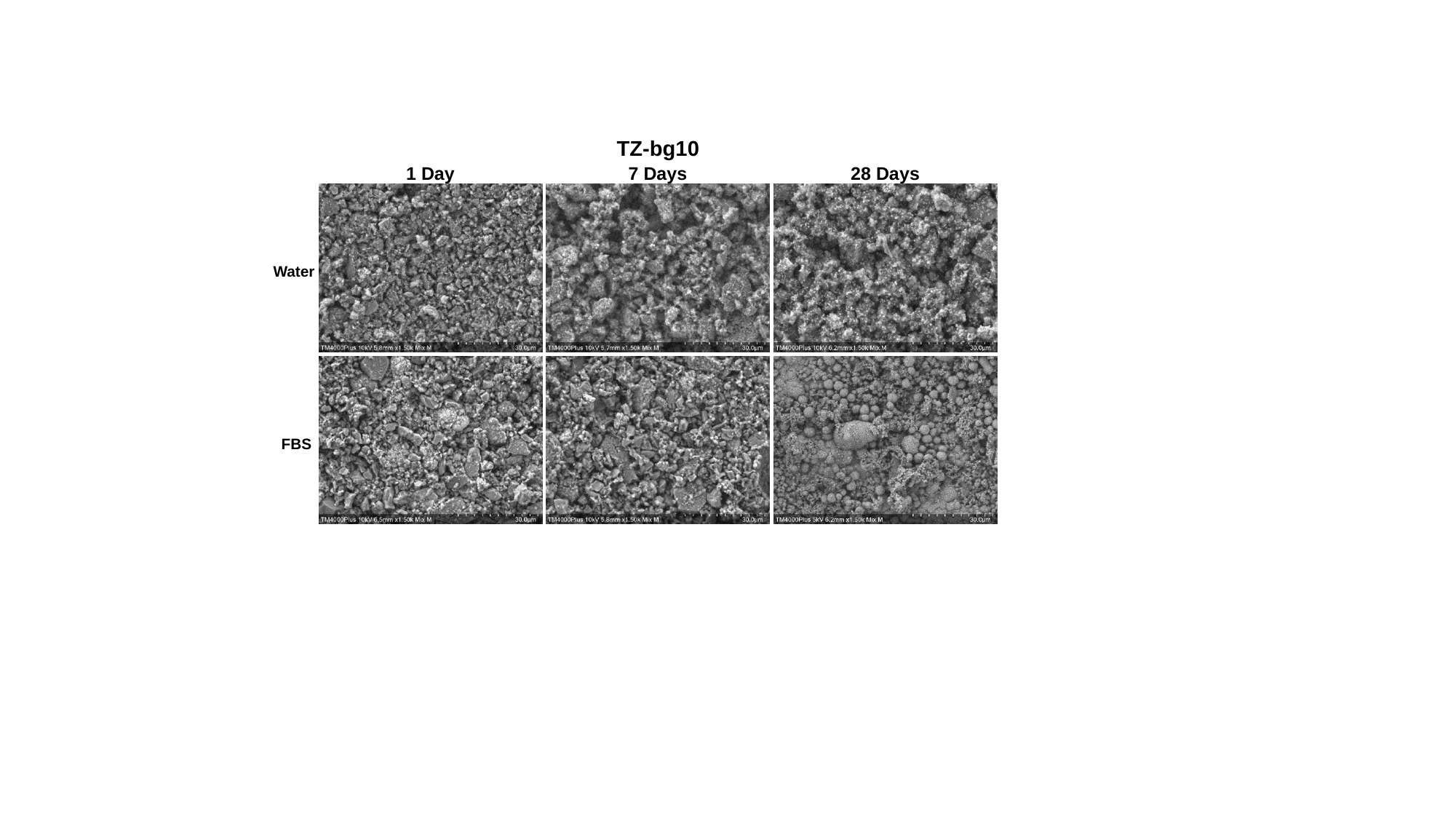

TZ-bg10
1 Day
7 Days
28 Days
Water
FBS

## Slide 6
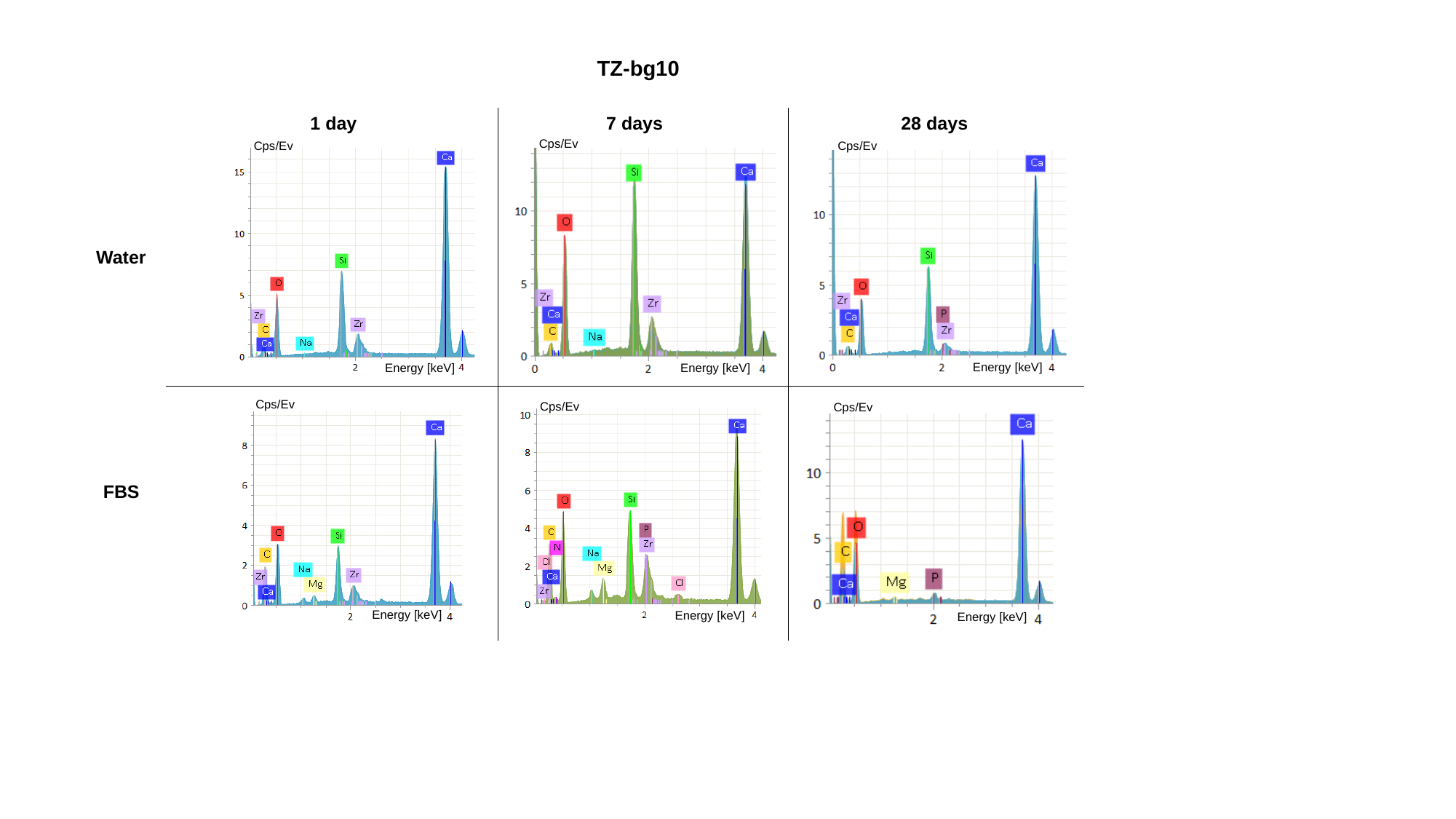

TZ-bg10
1 day
7 days
28 days
Cps/Ev
Energy [keV]
Cps/Ev
Energy [keV]
Cps/Ev
Energy [keV]
Water
Cps/Ev
Energy [keV]
Cps/Ev
Energy [keV]
Cps/Ev
Energy [keV]
FBS

## Slide 7
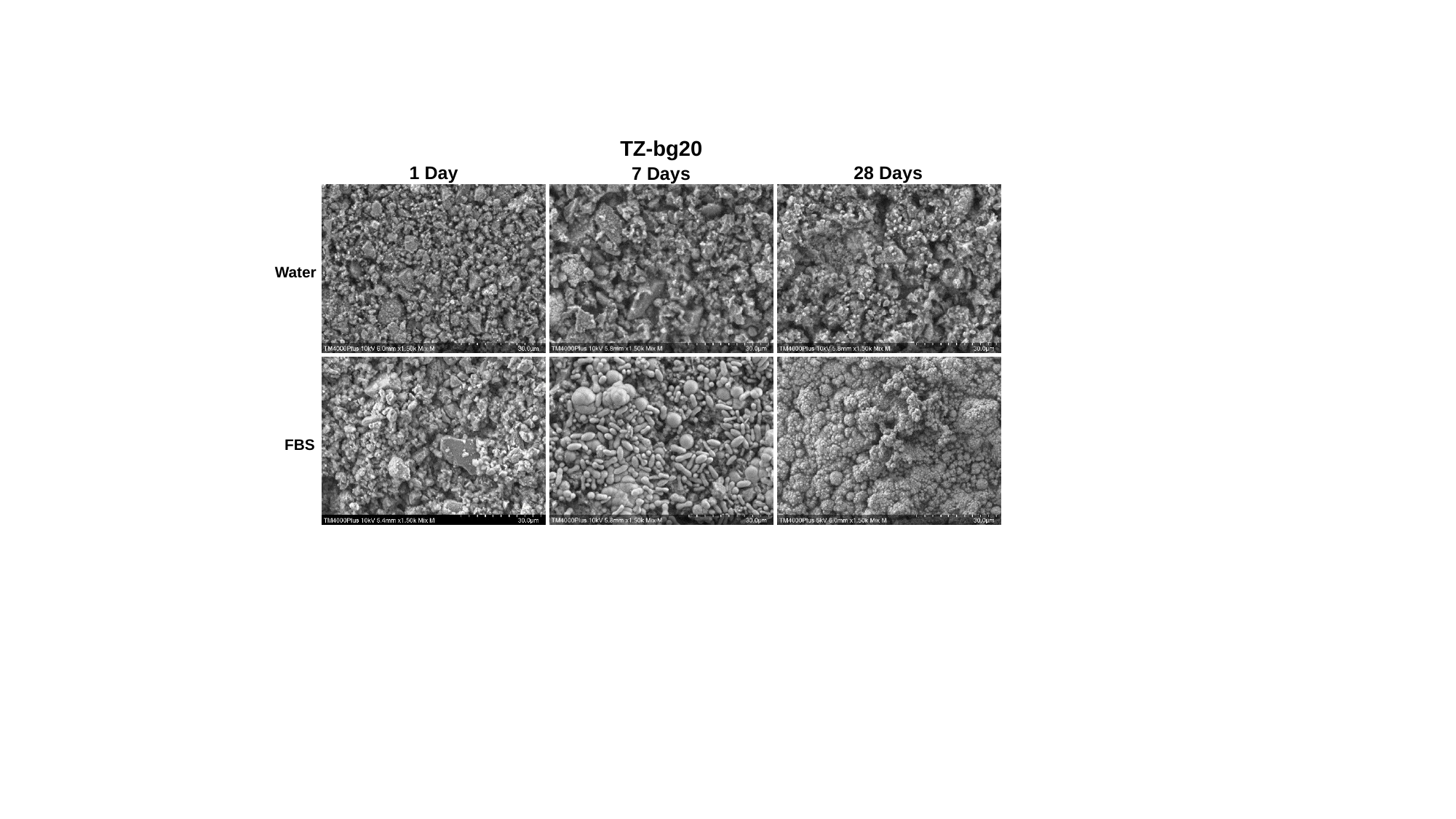

TZ-bg20
1 Day
28 Days
7 Days
Water
FBS

## Slide 8
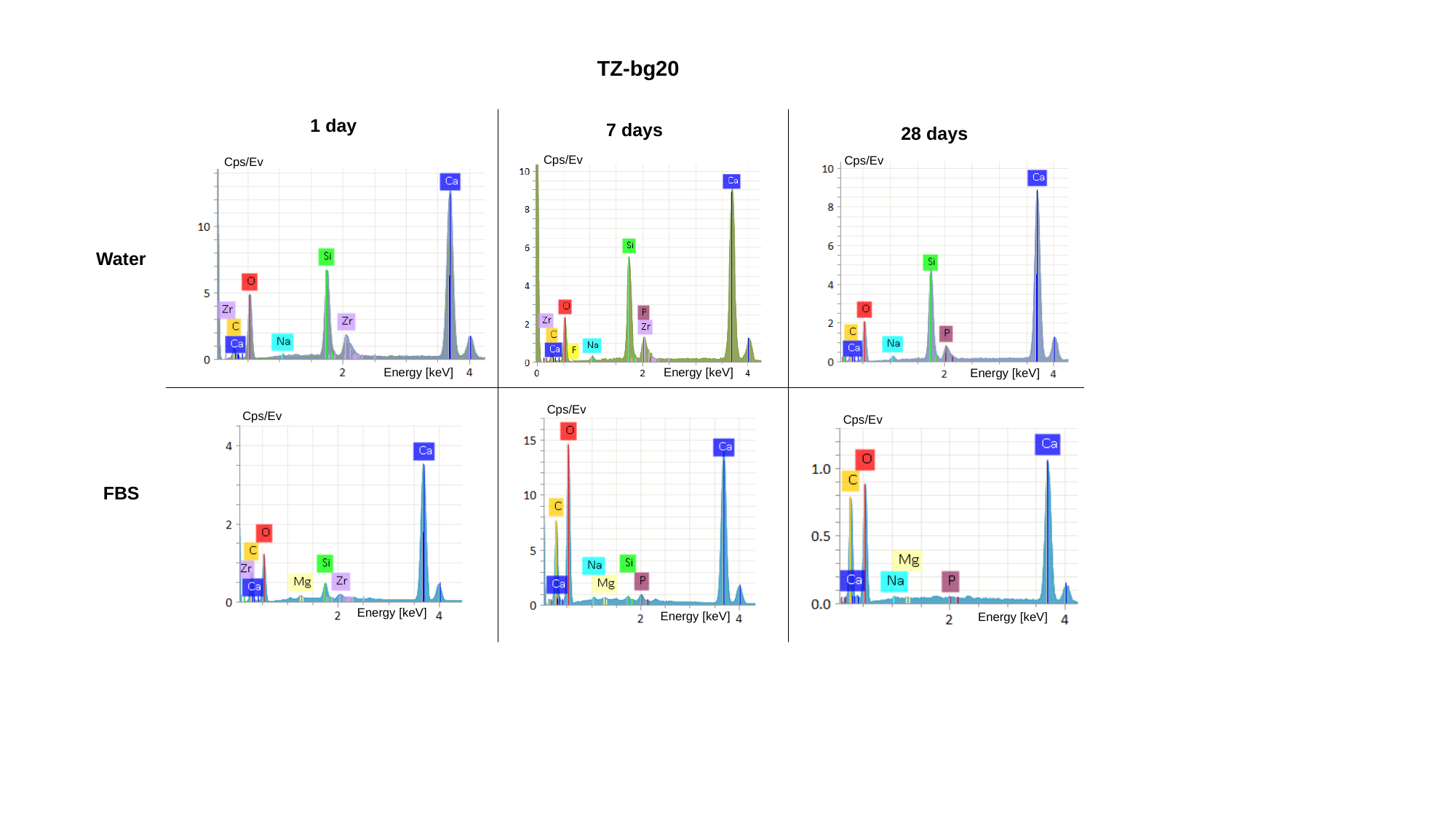

TZ-bg20
1 day
7 days
28 days
Cps/Ev
Energy [keV]
Cps/Ev
Energy [keV]
Cps/Ev
Energy [keV]
Water
Cps/Ev
Energy [keV]
Cps/Ev
Energy [keV]
Cps/Ev
Energy [keV]
FBS

## Slide 9
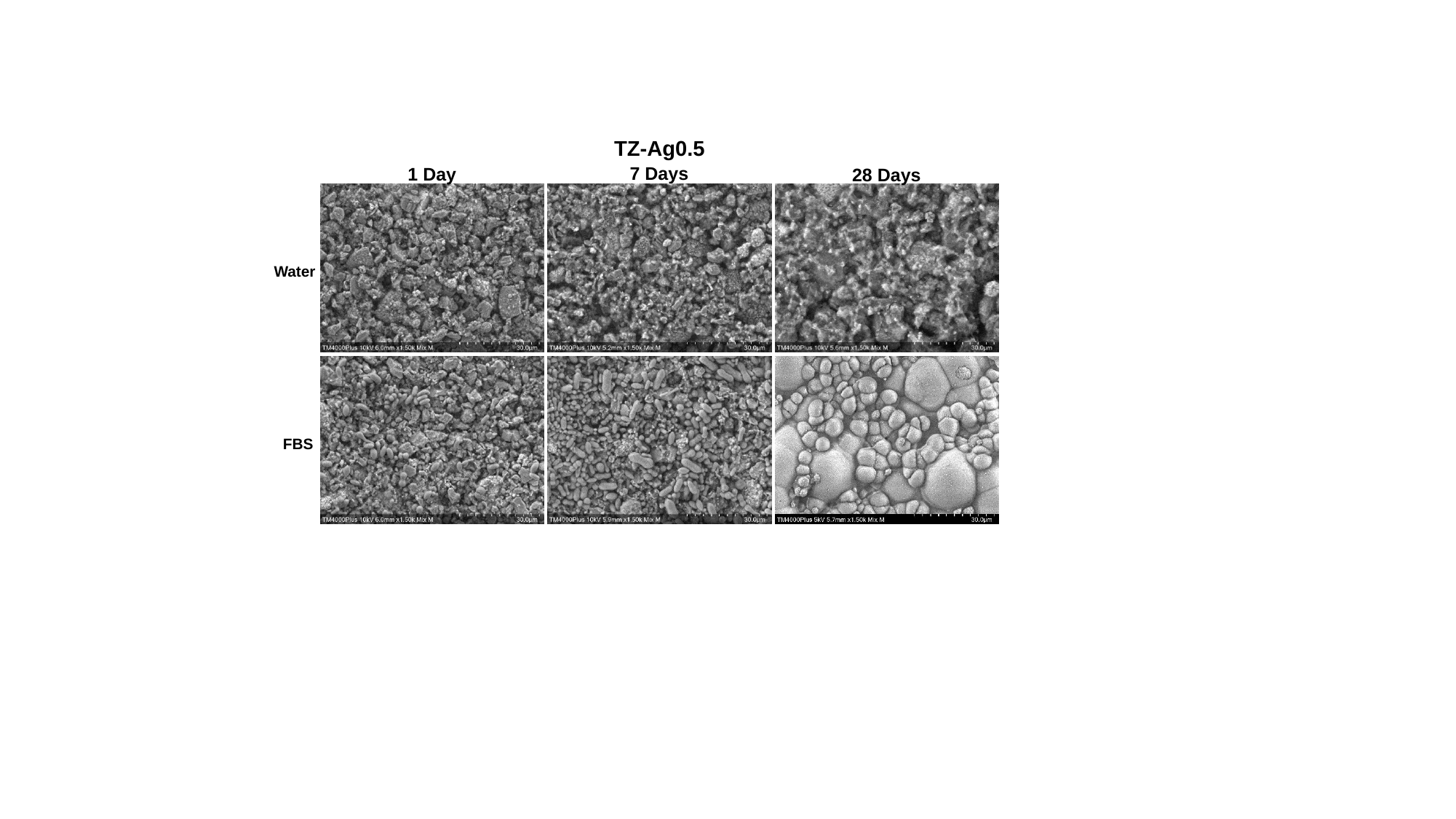

TZ-Ag0.5
7 Days
1 Day
28 Days
Water
FBS

## Slide 10
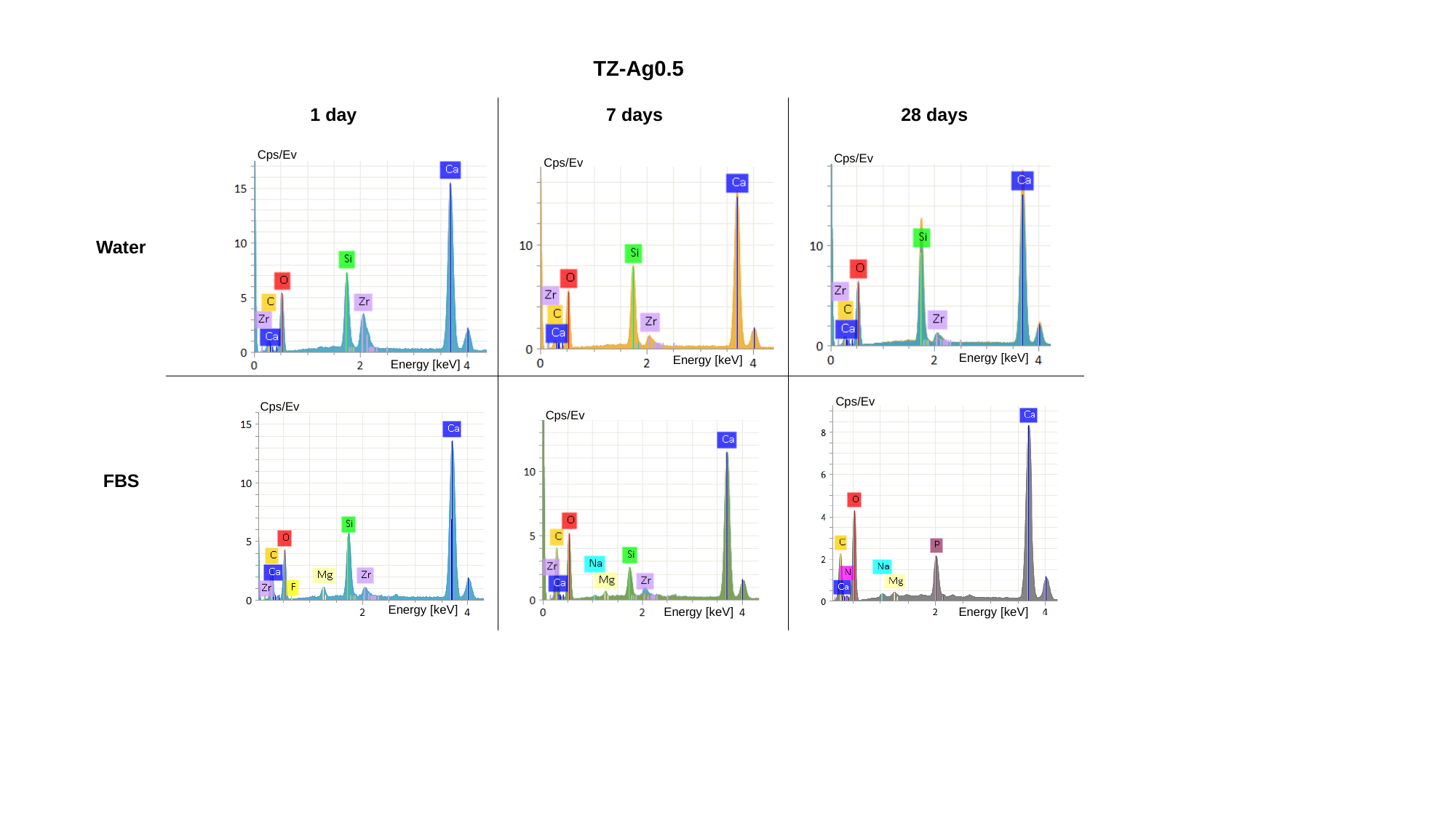

TZ-Ag0.5
1 day
7 days
28 days
Cps/Ev
Energy [keV]
Cps/Ev
Energy [keV]
Cps/Ev
Energy [keV]
Water
Cps/Ev
Energy [keV]
Cps/Ev
Energy [keV]
Cps/Ev
Energy [keV]
FBS

## Slide 11
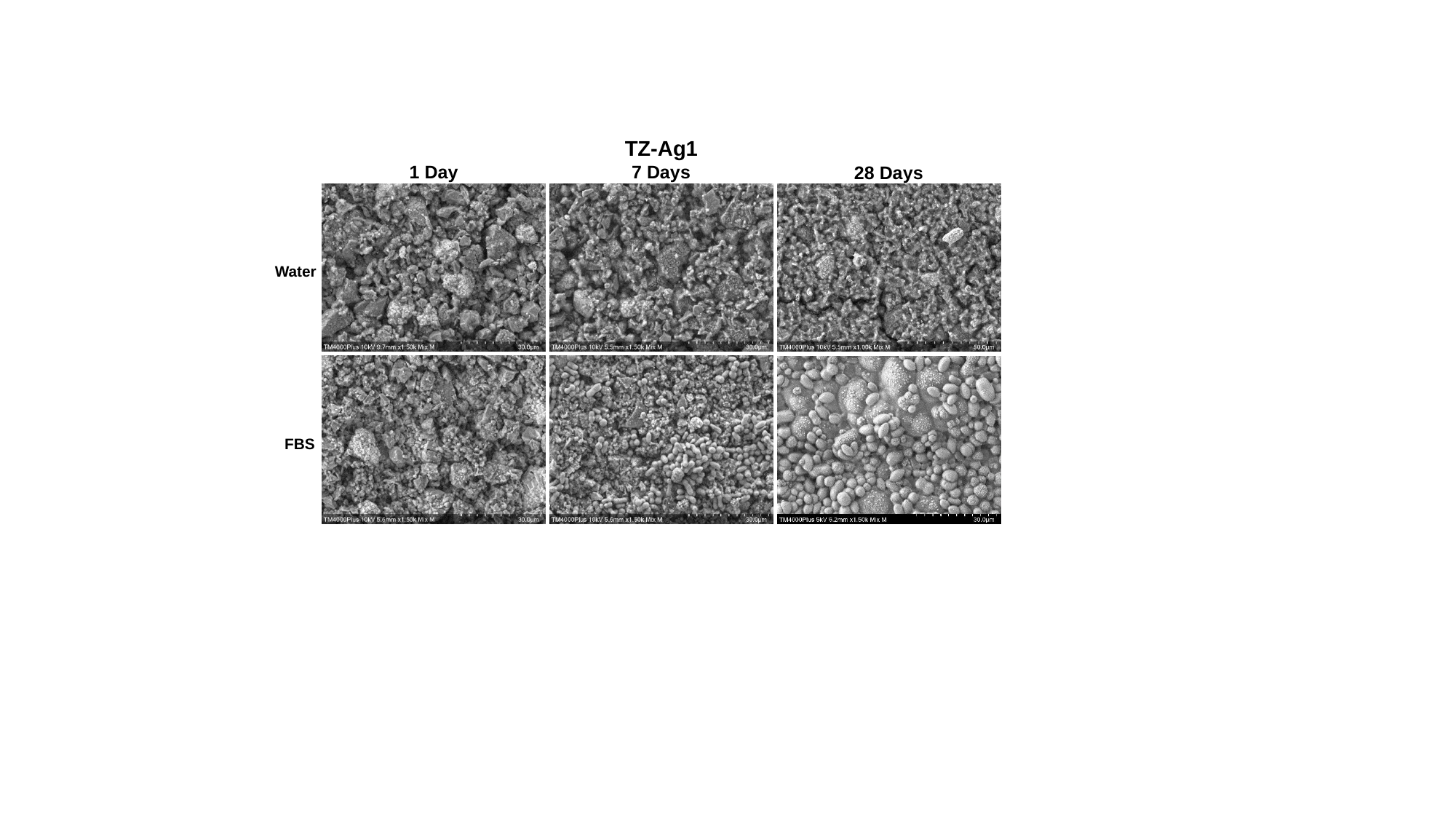

TZ-Ag1
7 Days
1 Day
28 Days
Water
FBS

## Slide 12
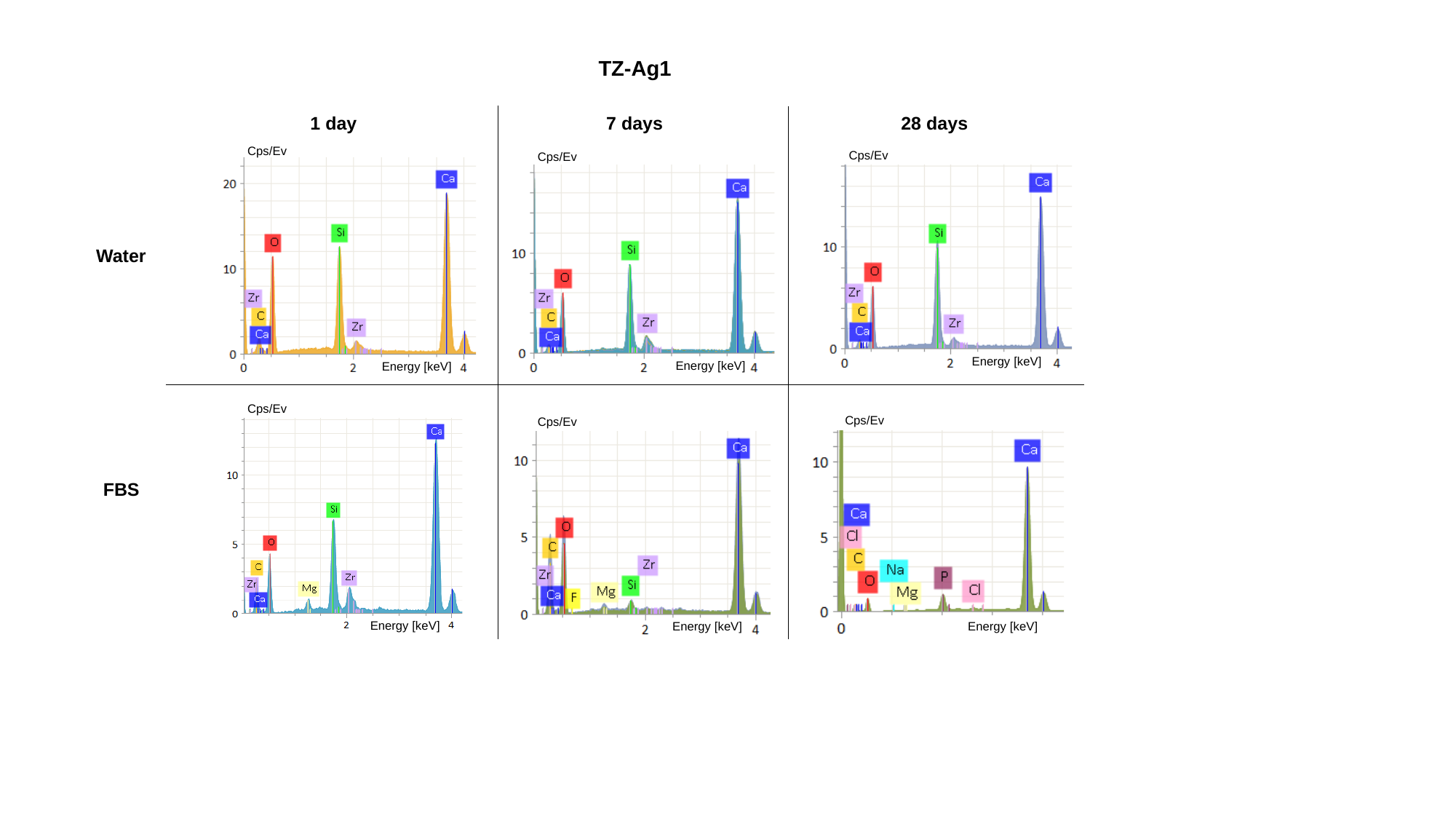

TZ-Ag1
1 day
7 days
28 days
Cps/Ev
Energy [keV]
Cps/Ev
Energy [keV]
Cps/Ev
Energy [keV]
Water
Cps/Ev
Energy [keV]
Cps/Ev
Energy [keV]
Cps/Ev
Energy [keV]
FBS
