# Supplementary material for: Surface characteristics and bacterial adhesion of endodontic cements
Source: Clin Oral Investig. 2022 Aug 5;26(12):6995–7009. doi: 10.1007/s00784-022-04655-y (PMC9708781; doi:10.1007/s00784-022-04655-y)
Supplement: Supplementary file 2 — Supplementary file2 (PPTX 4.73 MB) [file 784_2022_4655_MOESM2_ESM.pptx]

## Slide 1
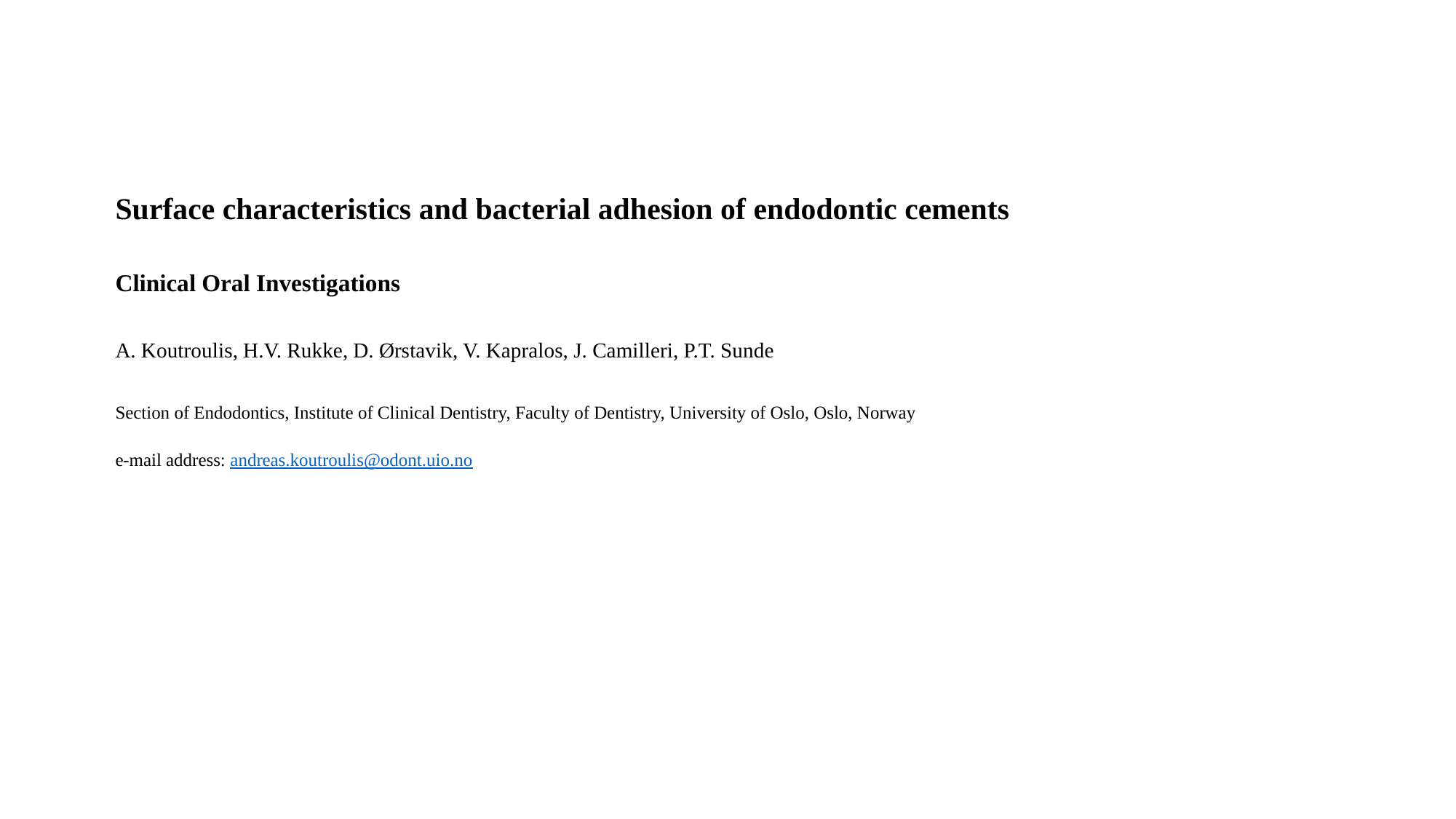

Surface characteristics and bacterial adhesion of endodontic cements
Clinical Oral Investigations
A. Koutroulis, H.V. Rukke, D. Ørstavik, V. Kapralos, J. Camilleri, P.T. Sunde
Section of Endodontics, Institute of Clinical Dentistry, Faculty of Dentistry, University of Oslo, Oslo, Norway
e-mail address: andreas.koutroulis@odont.uio.no

## Slide 2
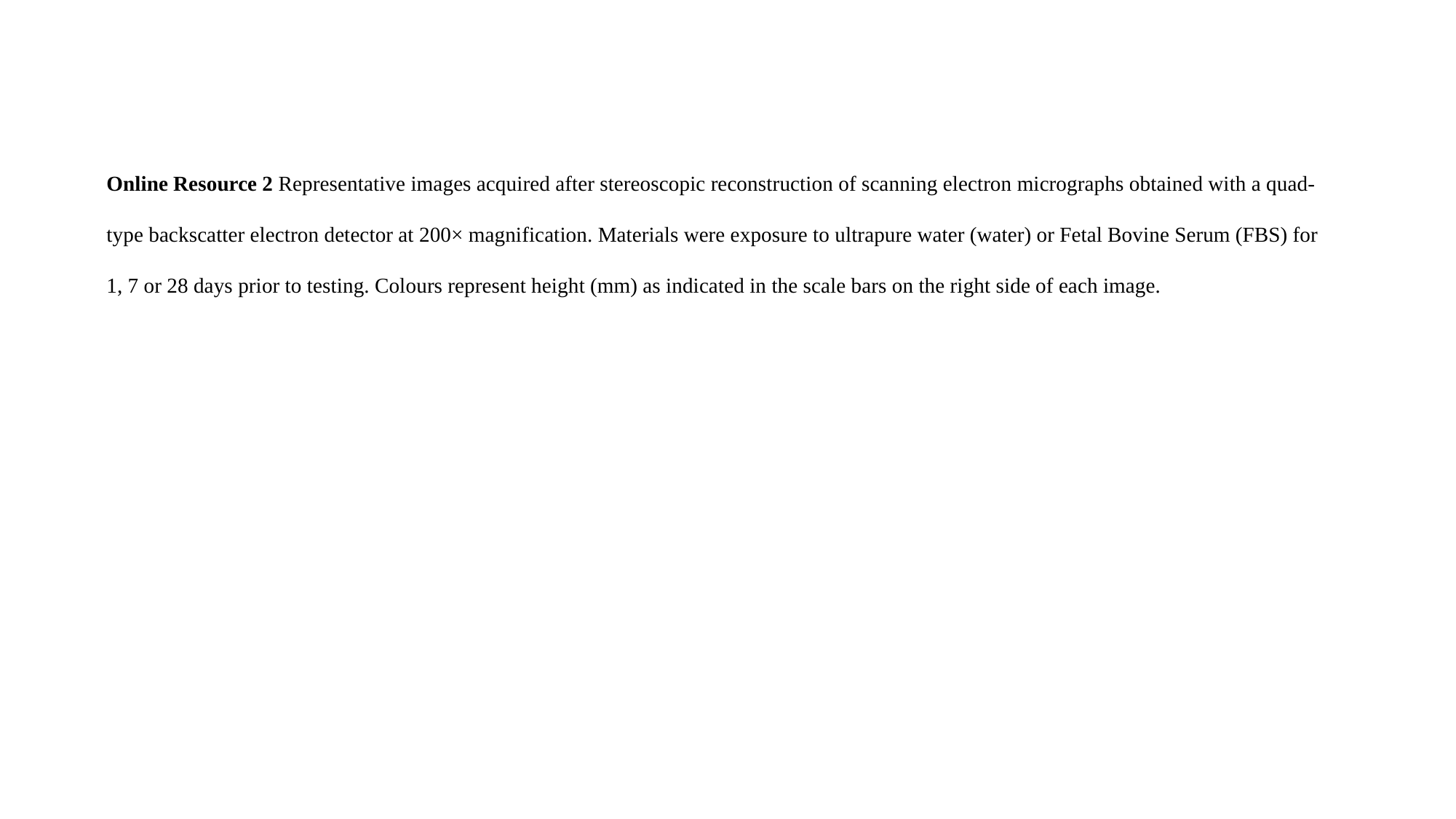

# Online Resource 2 Representative images acquired after stereoscopic reconstruction of scanning electron micrographs obtained with a quad-type backscatter electron detector at 200× magnification. Materials were exposure to ultrapure water (water) or Fetal Bovine Serum (FBS) for 1, 7 or 28 days prior to testing. Colours represent height (mm) as indicated in the scale bars on the right side of each image.

## Slide 3
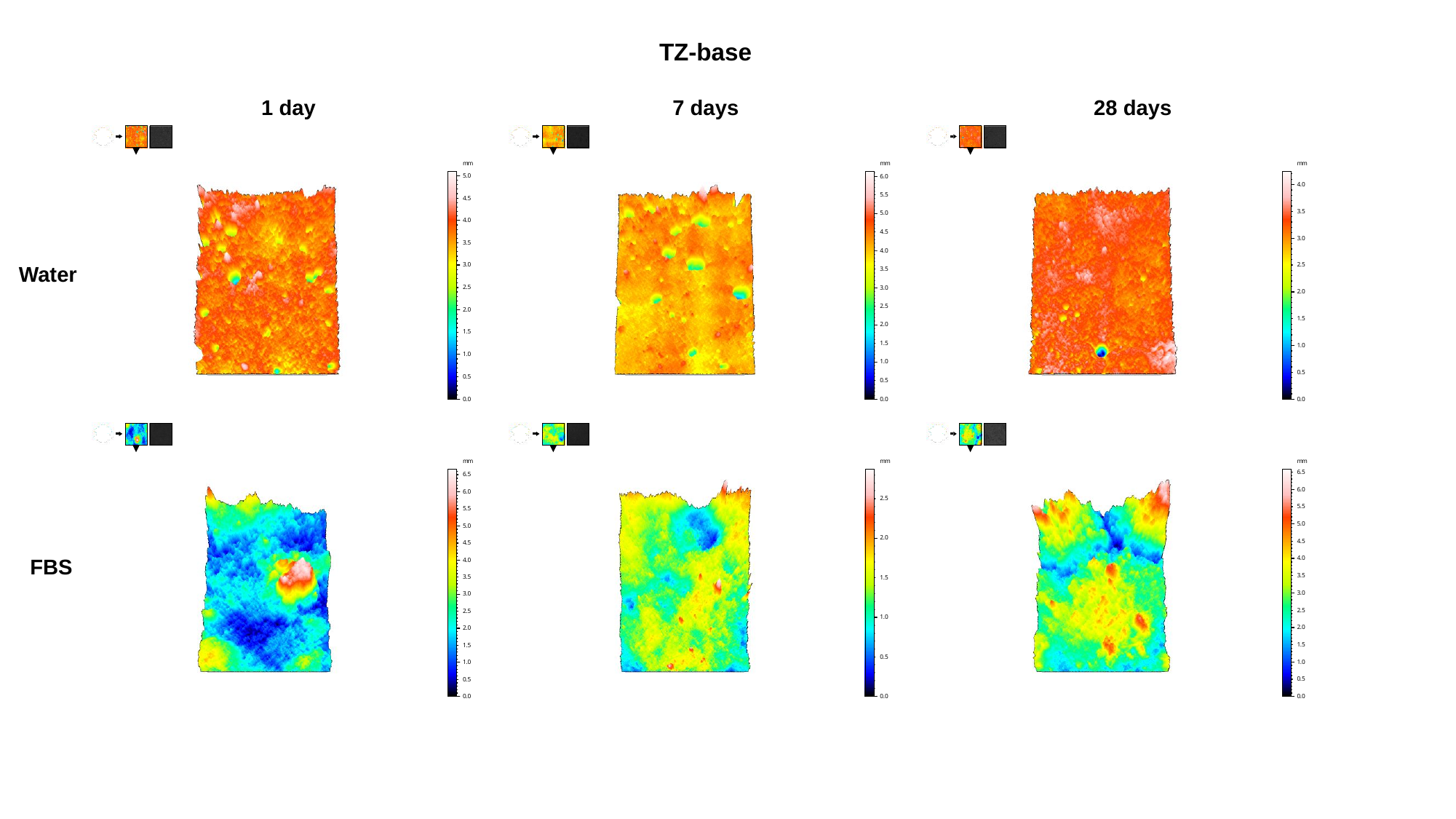

TZ-base
1 day
7 days
28 days
Water
FBS

## Slide 4
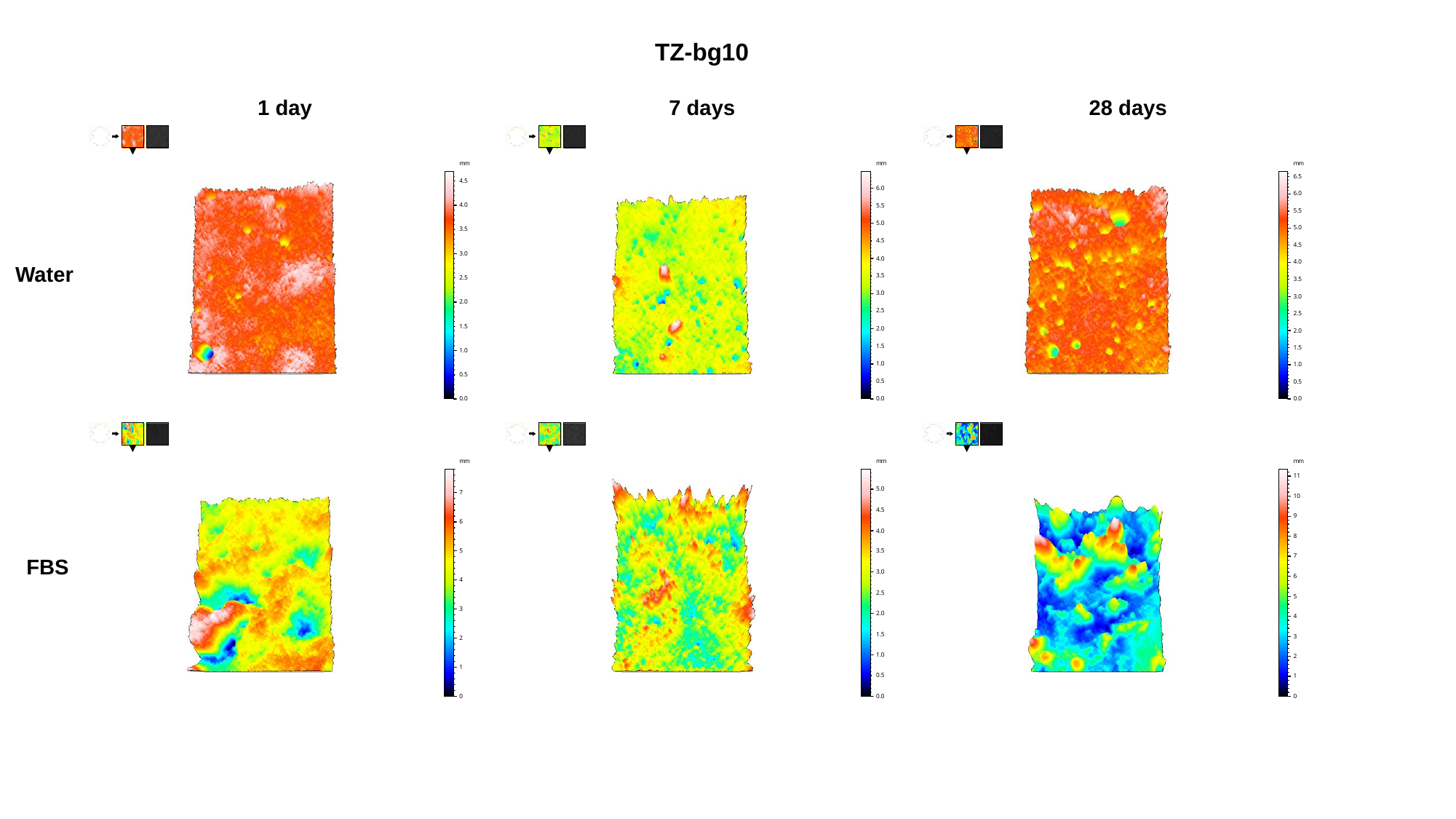

TZ-bg10
1 day
7 days
28 days
Water
FBS

## Slide 5
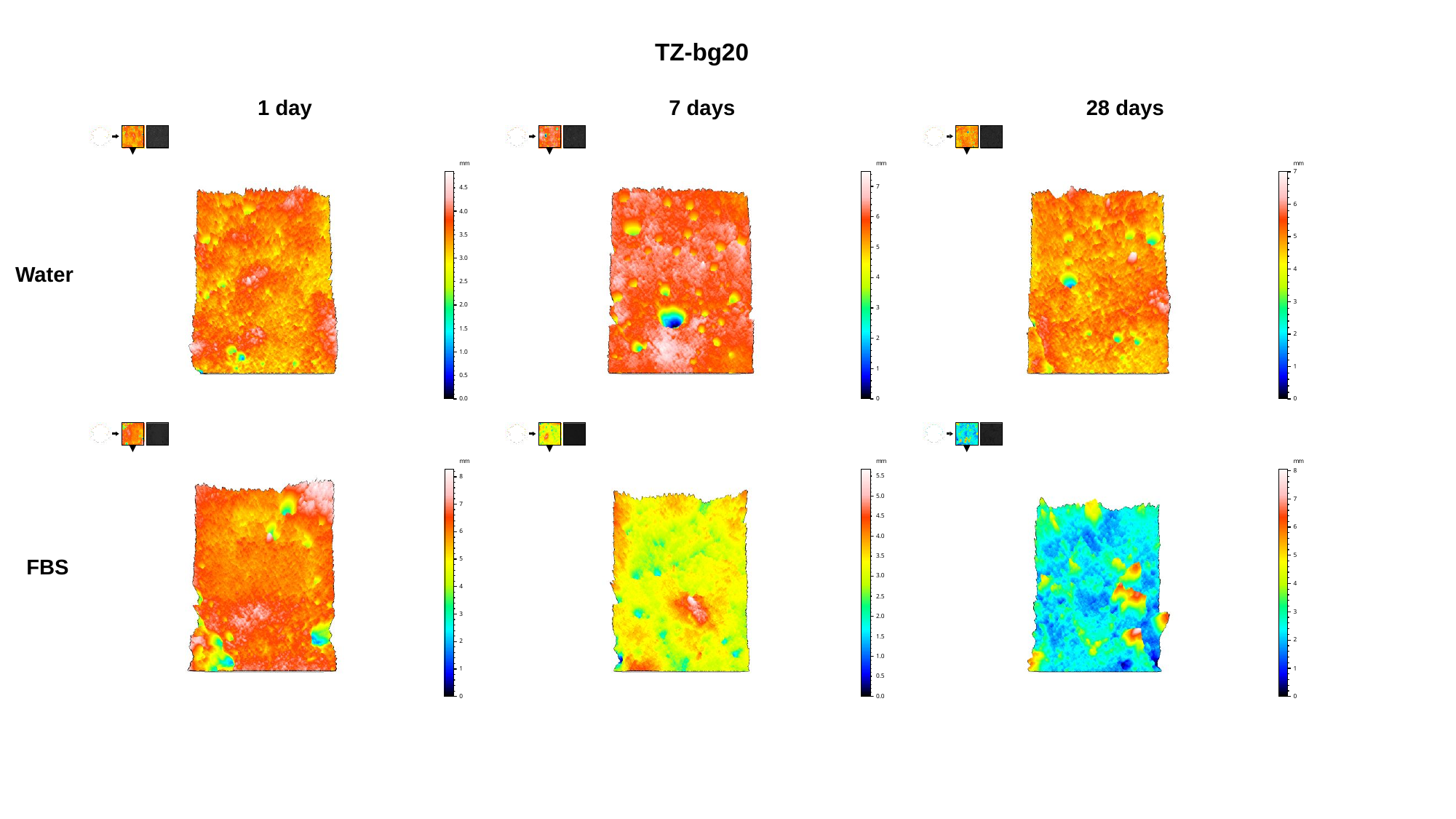

TZ-bg20
1 day
7 days
28 days
Water
FBS

## Slide 6
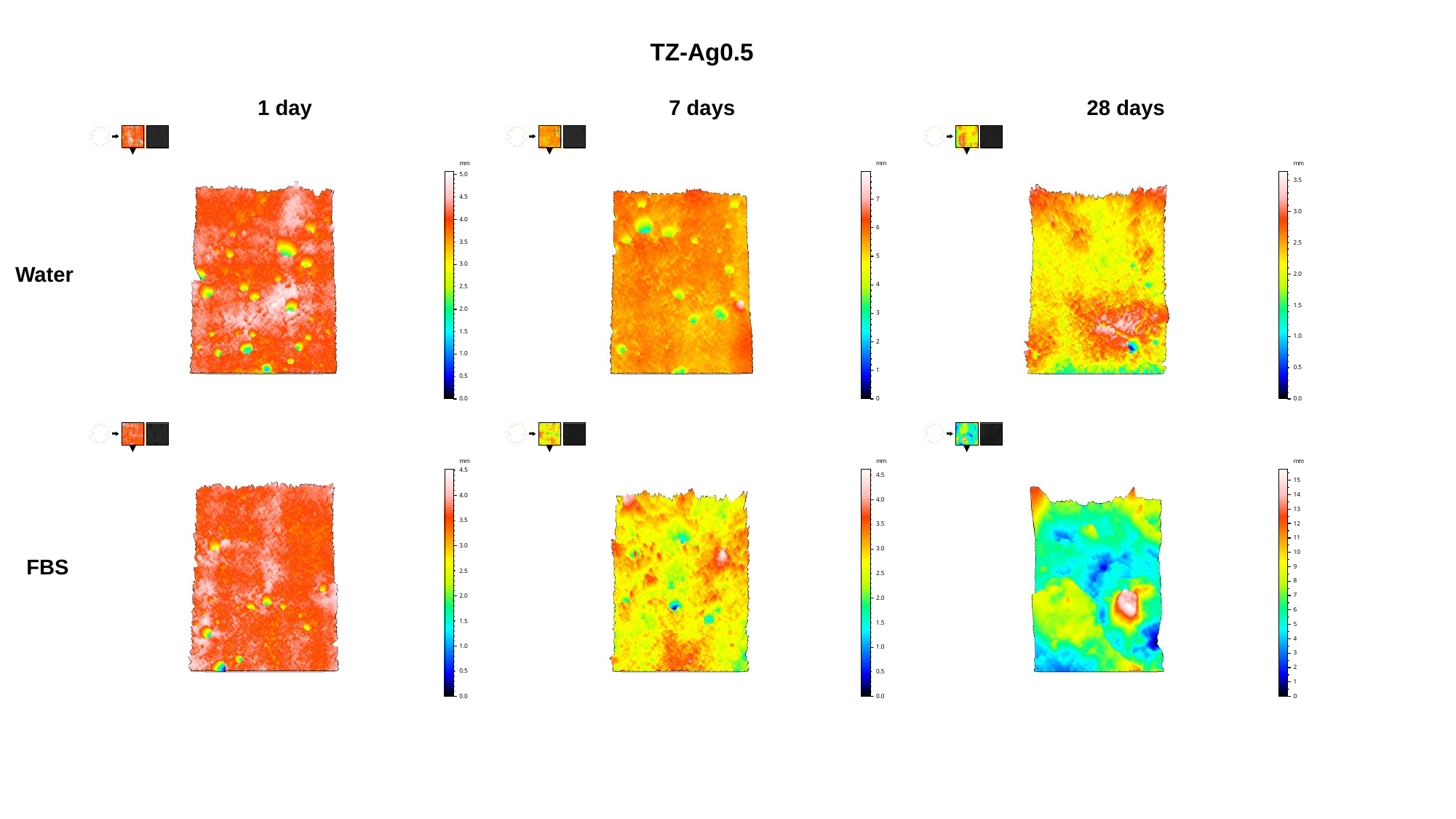

TZ-Ag0.5
1 day
7 days
28 days
Water
FBS

## Slide 7
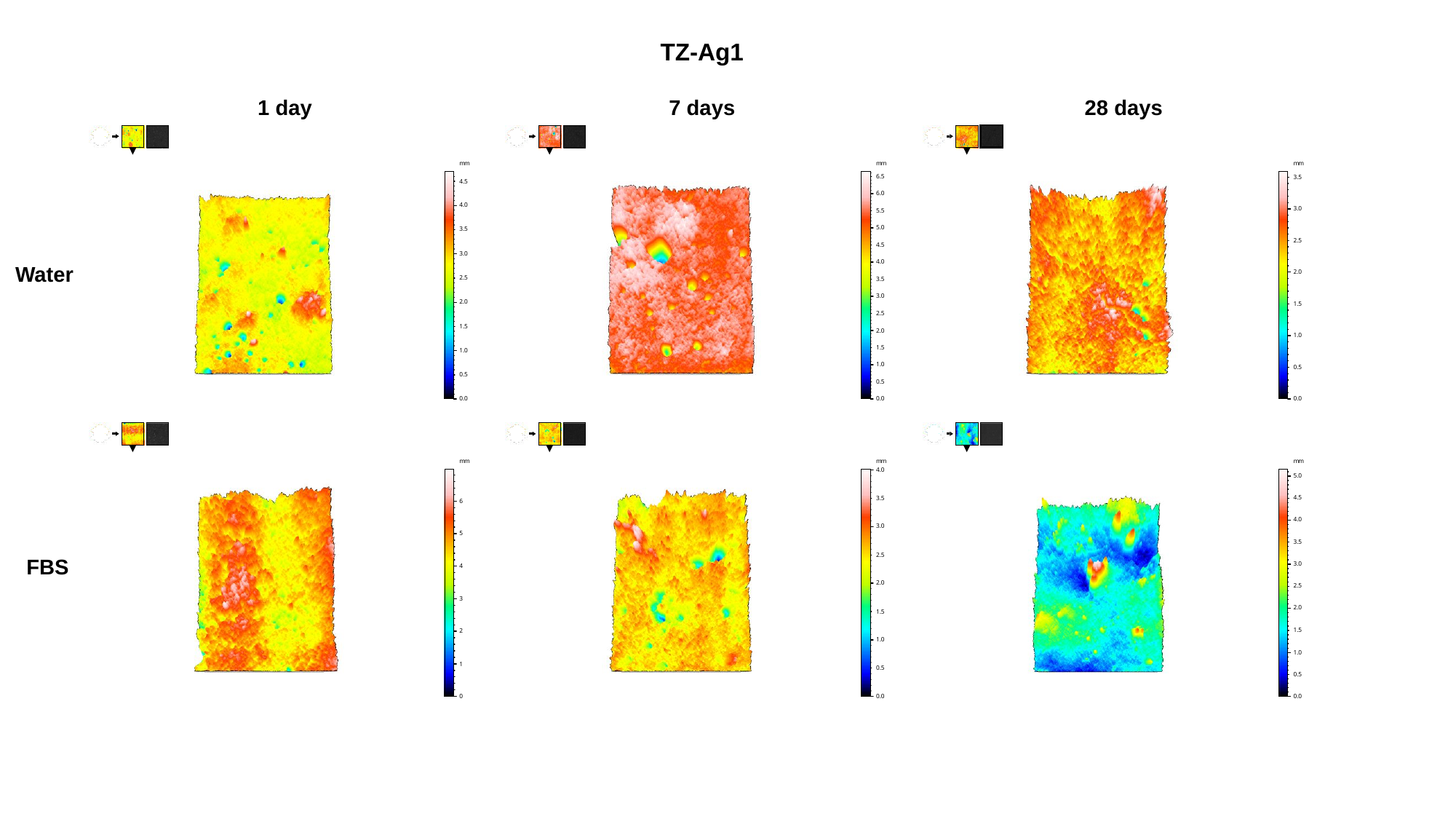

TZ-Ag1
1 day
7 days
28 days
Water
FBS

## Slide 8
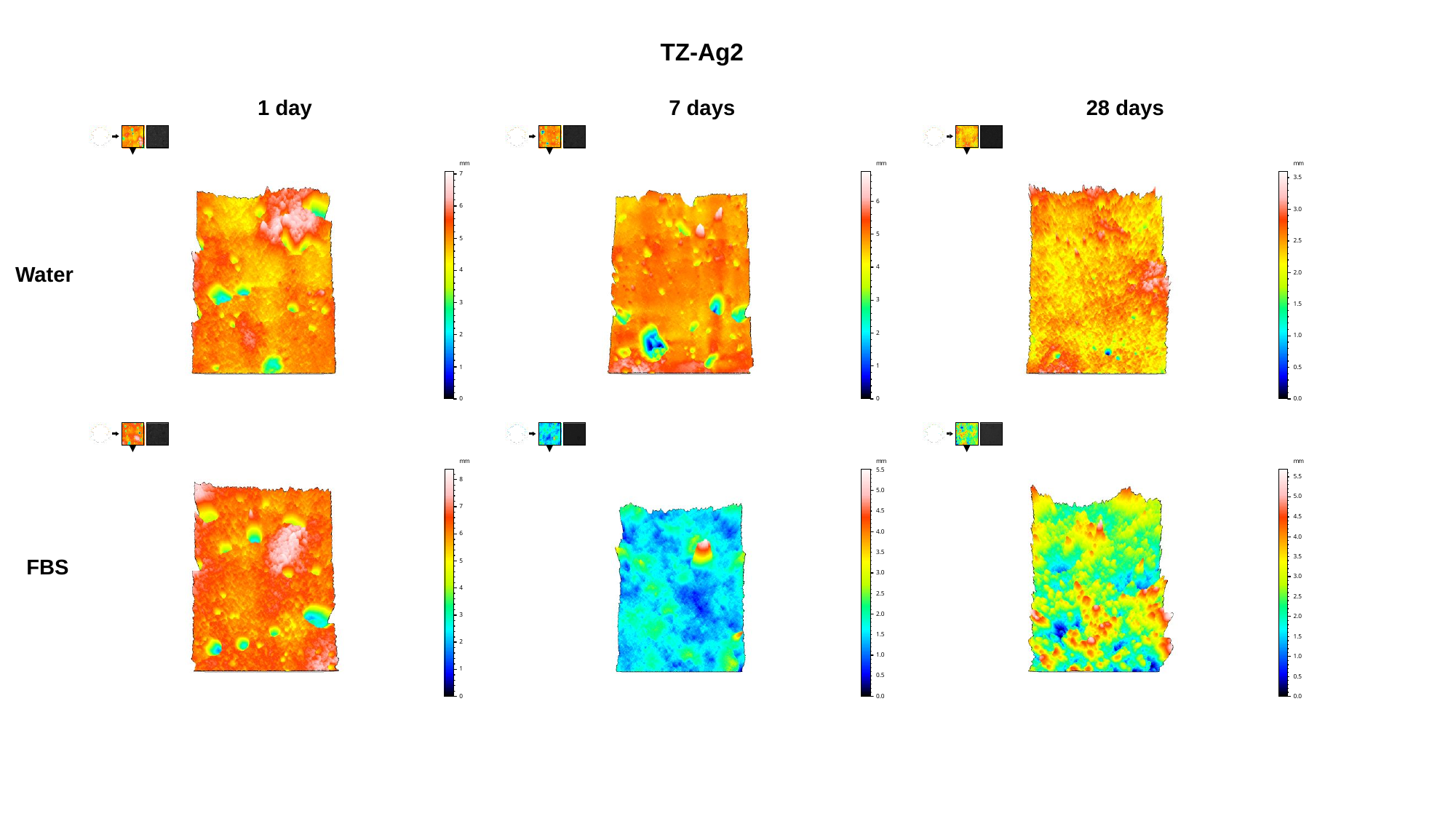

TZ-Ag2
1 day
7 days
28 days
Water
FBS

## Slide 9
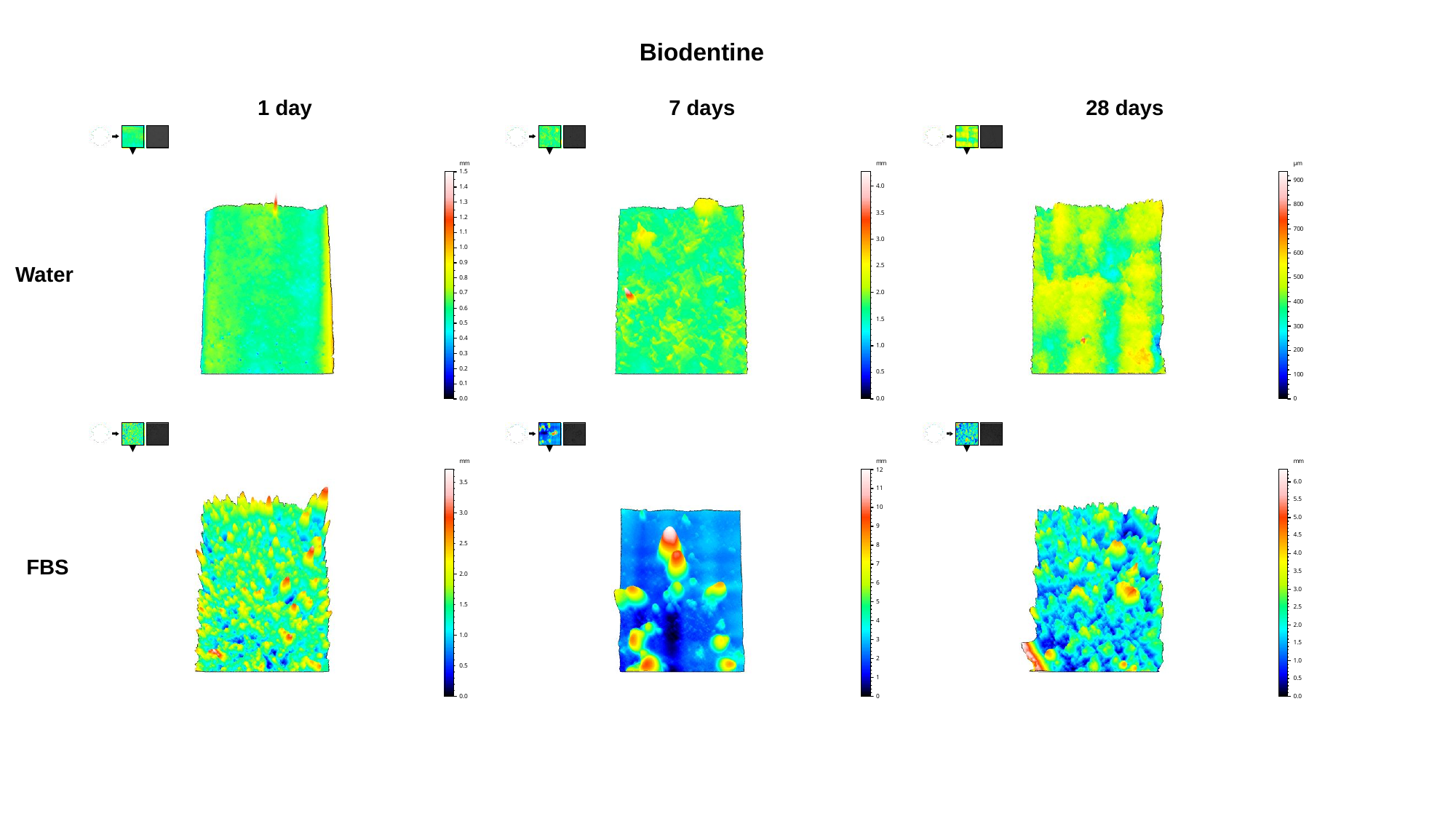

Biodentine
1 day
7 days
28 days
Water
FBS

## Slide 10
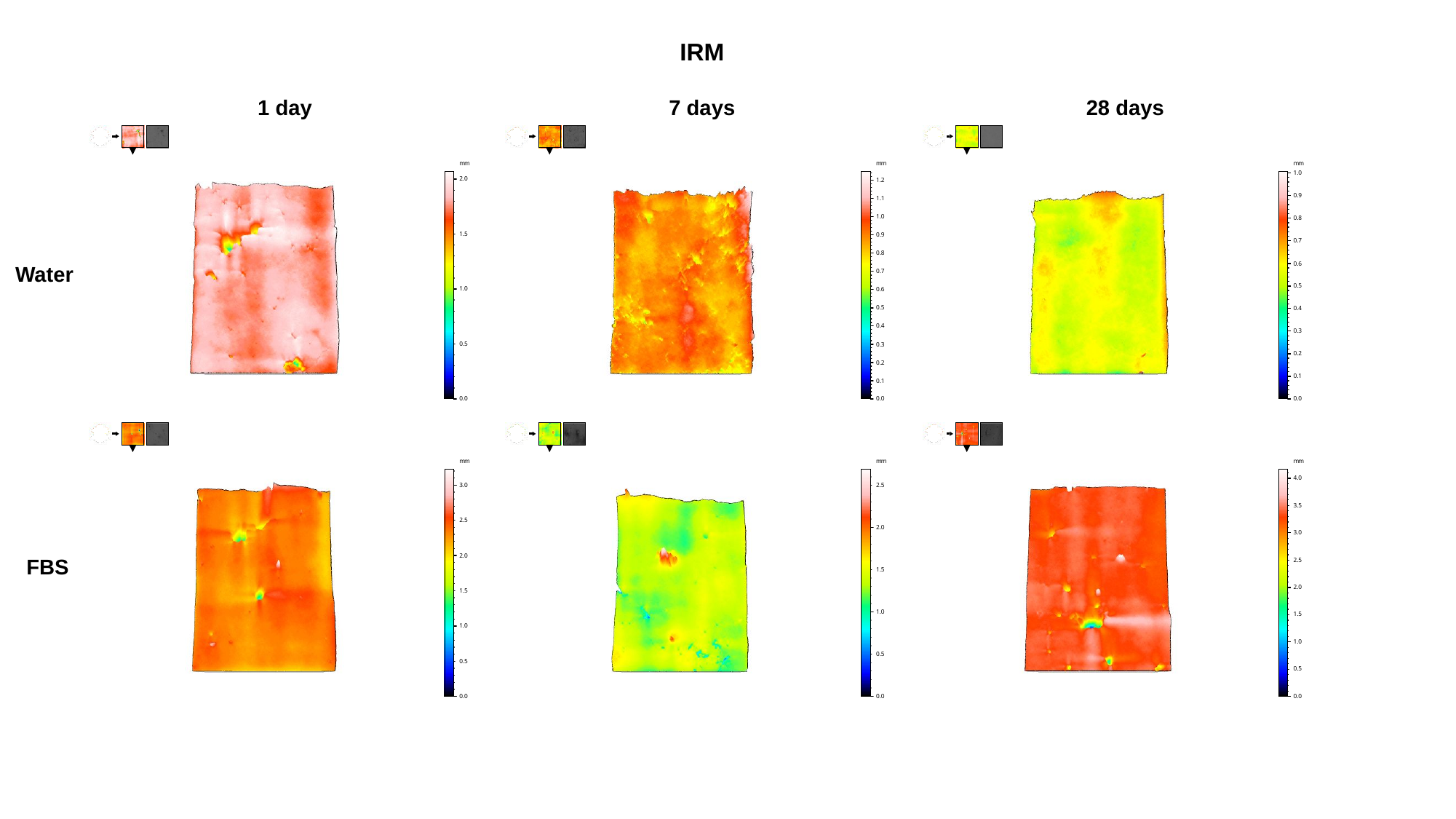

IRM
1 day
7 days
28 days
Water
FBS
