# Supplementary material for: Surface characteristics and bacterial adhesion of endodontic cements
Source: Clin Oral Investig. 2022 Aug 5;26(12):6995–7009. doi: 10.1007/s00784-022-04655-y (PMC9708781; doi:10.1007/s00784-022-04655-y)
Supplement: Supplementary file 3 — Supplementary file3 (PPTX 272 KB) [file 784_2022_4655_MOESM3_ESM.pptx]

## Slide 1
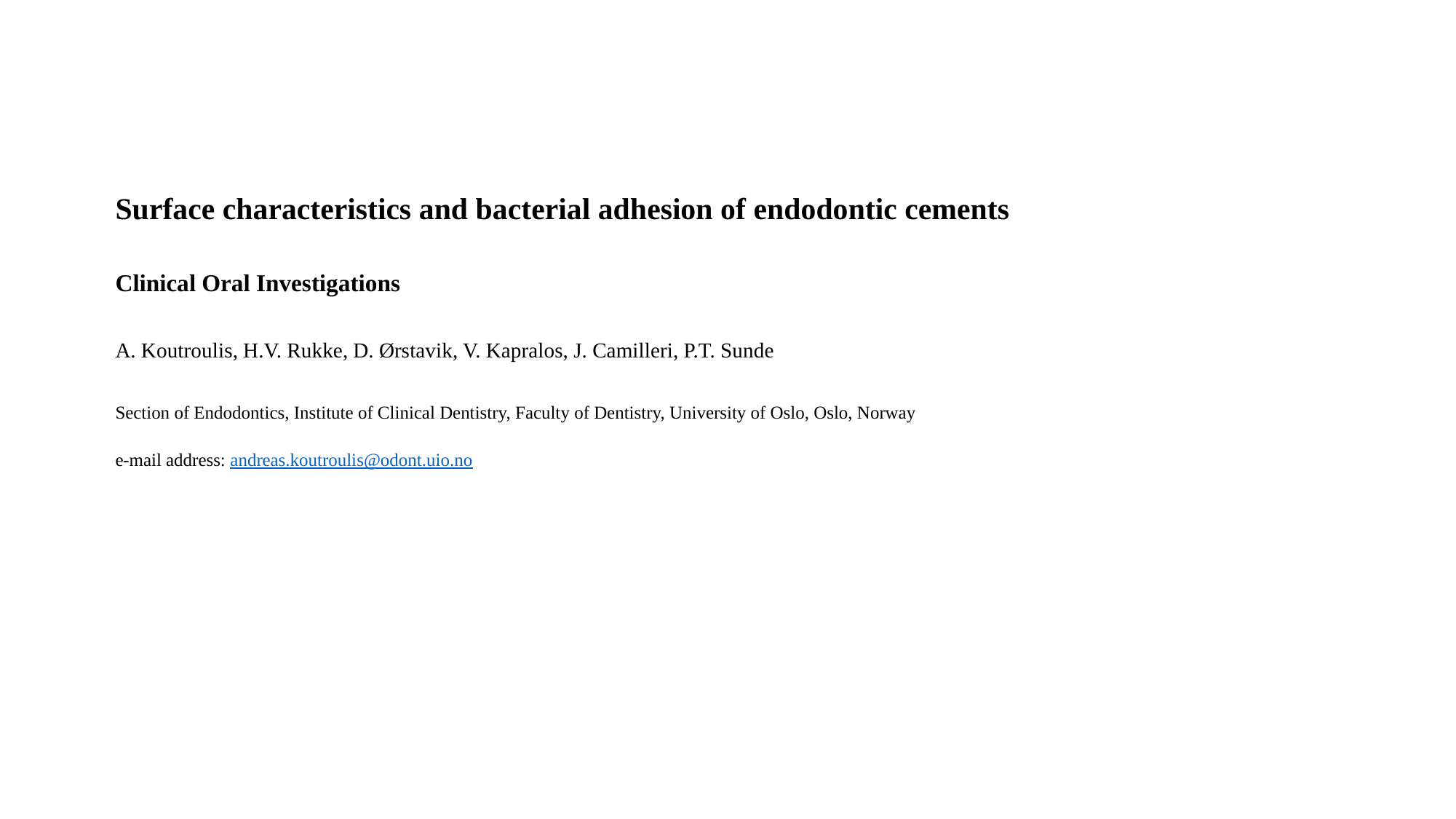

Surface characteristics and bacterial adhesion of endodontic cements
Clinical Oral Investigations
A. Koutroulis, H.V. Rukke, D. Ørstavik, V. Kapralos, J. Camilleri, P.T. Sunde
Section of Endodontics, Institute of Clinical Dentistry, Faculty of Dentistry, University of Oslo, Oslo, Norway
e-mail address: andreas.koutroulis@odont.uio.no

## Slide 2
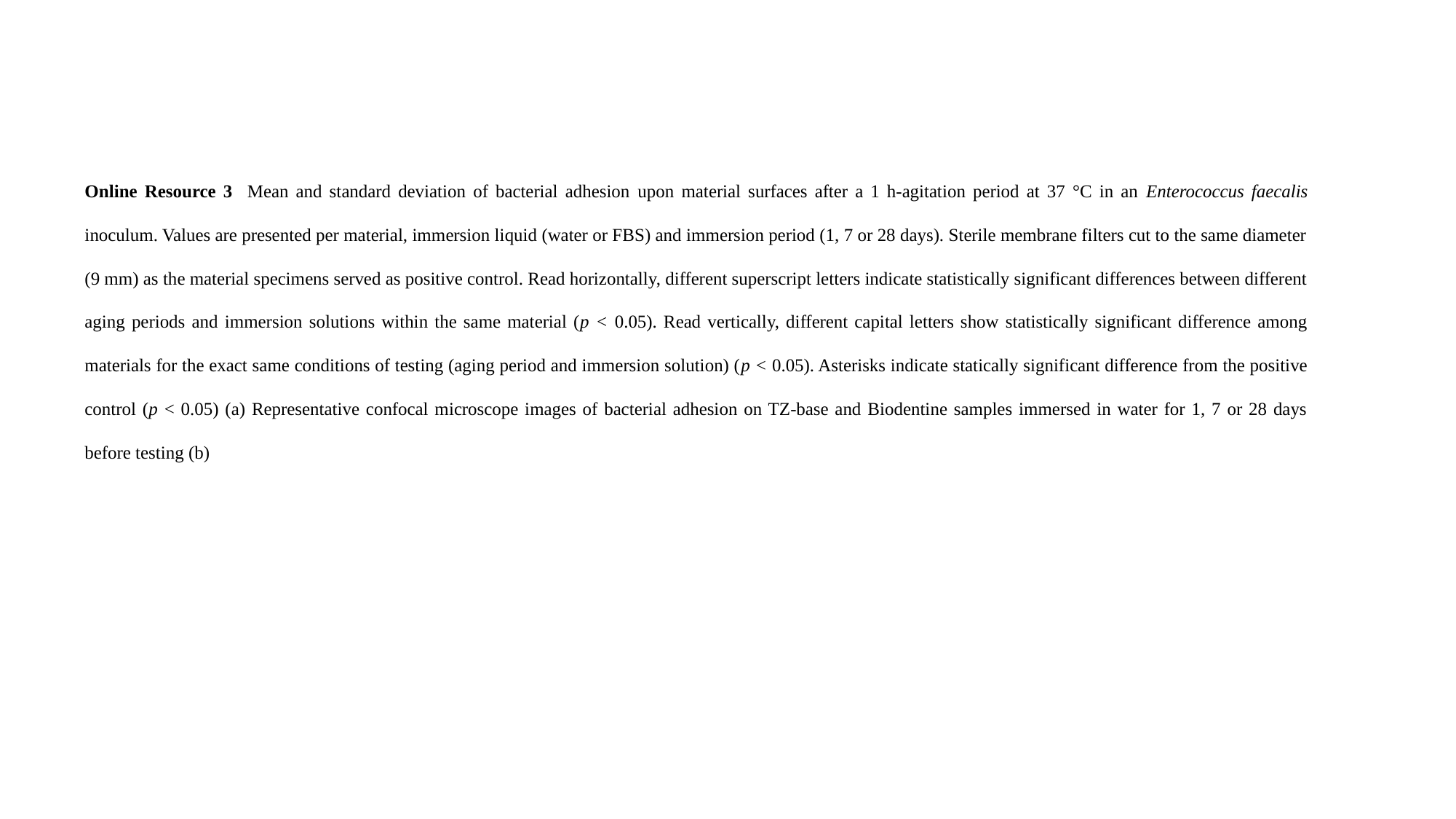

Online Resource 3 Mean and standard deviation of bacterial adhesion upon material surfaces after a 1 h-agitation period at 37 °C in an Enterococcus faecalis inoculum. Values are presented per material, immersion liquid (water or FBS) and immersion period (1, 7 or 28 days). Sterile membrane filters cut to the same diameter (9 mm) as the material specimens served as positive control. Read horizontally, different superscript letters indicate statistically significant differences between different aging periods and immersion solutions within the same material (p < 0.05). Read vertically, different capital letters show statistically significant difference among materials for the exact same conditions of testing (aging period and immersion solution) (p < 0.05). Asterisks indicate statically significant difference from the positive control (p < 0.05) (a) Representative confocal microscope images of bacterial adhesion on TZ-base and Biodentine samples immersed in water for 1, 7 or 28 days before testing (b)

## Slide 3
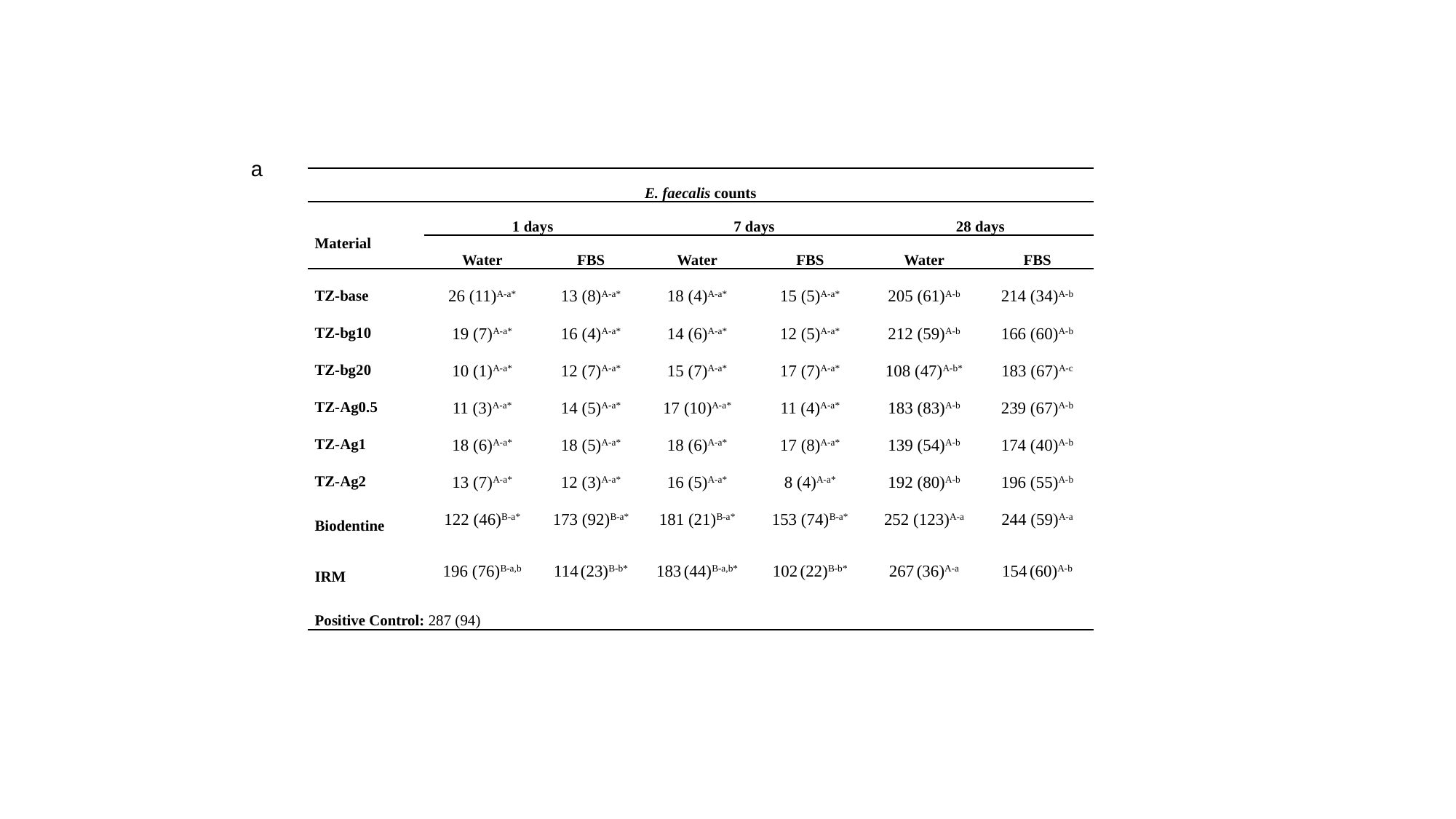

a
| E. faecalis counts | | | | | | |
| --- | --- | --- | --- | --- | --- | --- |
| Material | 1 days | | 7 days | | 28 days | |
| | Water | FBS | Water | FBS | Water | FBS |
| TZ-base | 26 (11)A-a\* | 13 (8)A-a\* | 18 (4)A-a\* | 15 (5)A-a\* | 205 (61)A-b | 214 (34)A-b |
| TZ-bg10 | 19 (7)A-a\* | 16 (4)A-a\* | 14 (6)A-a\* | 12 (5)A-a\* | 212 (59)A-b | 166 (60)A-b |
| TZ-bg20 | 10 (1)A-a\* | 12 (7)A-a\* | 15 (7)A-a\* | 17 (7)A-a\* | 108 (47)A-b\* | 183 (67)A-c |
| TZ-Ag0.5 | 11 (3)A-a\* | 14 (5)A-a\* | 17 (10)A-a\* | 11 (4)A-a\* | 183 (83)A-b | 239 (67)A-b |
| TZ-Ag1 | 18 (6)A-a\* | 18 (5)A-a\* | 18 (6)A-a\* | 17 (8)A-a\* | 139 (54)A-b | 174 (40)A-b |
| TZ-Ag2 | 13 (7)A-a\* | 12 (3)A-a\* | 16 (5)A-a\* | 8 (4)A-a\* | 192 (80)A-b | 196 (55)A-b |
| Biodentine | 122 (46)B-a\* | 173 (92)B-a\* | 181 (21)B-a\* | 153 (74)B-a\* | 252 (123)A-a | 244 (59)A-a |
| IRM | 196 (76)B-a,b | 114 (23)B-b\* | 183 (44)B-a,b\* | 102 (22)B-b\* | 267 (36)A-a | 154 (60)A-b |
| Positive Control: 287 (94) | | | | | | |

## Slide 4
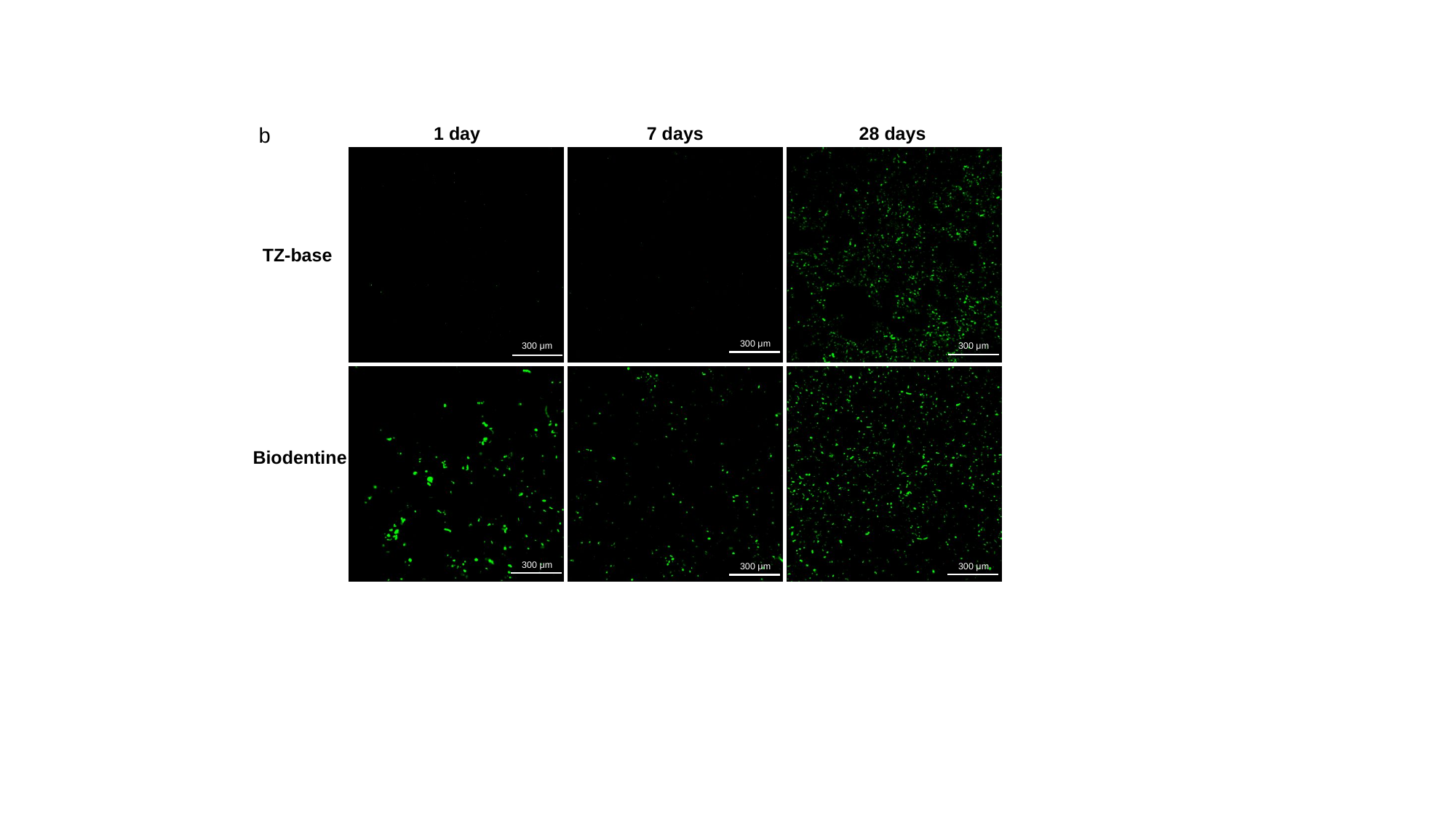

1 day
28 days
7 days
TZ-base
Biodentine
b
300 μm
300 μm
300 μm
300 μm
300 μm
300 μm
